# Trends for Proton Transport Activity and Stability in Turnbull’s Blue Analogues: Theory and Experiments

**DOI:** 10.1021/acs.chemmater.5c03404

**Published:** 2026-06-01

**Authors:** Mengru Li, Rui Wang, Pen-Yeh Yen, Anneke Moeller, Reagan Shippy, Katelyn H. Michael, Victor M. Zavala, Song Jin, Manos Mavrikakis

**Affiliations:** † Department of Chemical and Biological Engineering, 489540 University of Wisconsin−Madison, Madison, Wisconsin 53706, United States; ‡ Department of Chemistry, University of Wisconsin−Madison, Madison, Wisconsin 53706, United States

## Abstract

Prussian Blue analogues (PBAs) are promising materials for energy storage and conversion because their robust open-framework structures enable efficient ion intercalation. Among them, Turnbull’s Blue analogues (TBAs), a subclass featuring ferrocyanide vacancies, exhibit exceptional rate capability in acidic electrolytes by facilitating rapid proton transport. To optimize TBA performance, understanding how the metal identity influences both proton mobility and structural stability is essential. Here, we combined density functional theory (DFT) calculations with electrochemical characterization experiments to investigate the proton transport activity and stability of TBAs incorporating six metals (Cu, Zn, Mn, Fe, Co, and Ni) under acidic conditions. Our theoretical analysis revealed that the inclusion of Mn, Fe, Co, and Ni enhances proton transport via electron spin-flipping at low-spin Fe sites. Furthermore, the presence of ferromagnetic metals promotes the reduction of Fe ions located farther from dense ferrocyanide vacancy regions during proton intercalation, destabilizing the initial protonated configuration and lowering the reaction energy. Stability trends, assessed through Gibbs free energy changes for dissolution in acidic electrolytes, aligned with experimental capacity retention. Through this integrated study, Ni-TBA emerged as a promising candidate, combining high proton transport activity with superior stability, offering valuable guidance for the rational design of high-performance proton-ion battery materials for energy storage.

## Graphical abstract

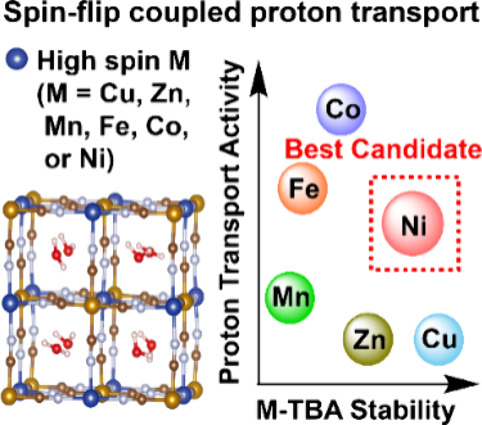

## 1 Introduction

Renewable energy sources such as solar and wind power and associated technologies are currently pursued to address the long-term global energy problem and decarbonize various industries.^1–3^ However, the intermittent nature of these renewable energy sources necessitates the development of efficient materials for energy storage and conversion. Redox reservoirs (RRs), a class of solid-state battery materials, can pair various electrochemical productions in decoupled electrochemical systems, enabling the flexible utilization of renewable electricity to produce a variety of value-added products.^4–7^ This is achieved through the intercalation and deintercalation of charged ions such as Na^+^, K^+^, H^+^, and OH^–^ in concert with the transfer of electrons,^8^ eliminating the need for sacrificial reactions that generate undesired byproducts in electrochemical synthesis. Moreover, integrating RRs into electrochemical systems provides flexibility in controlling electron transfer numbers, location, timing, reaction conditions, scale, and rates of paired half-reactions.^2,9,10^

Prussian blue analogues (PBAs) are known for their cost-effectiveness, straightforward synthesis methods, and open framework structures that facilitate the reversible intercalation and deintercalation of various ions.^11^ These features render PBAs suitable not only as electrode materials^12–15^ for batteries and supercapacitors^16,17^ but also as effective RRs in decoupled electrochemical systems.^2,5,18,19^ Among PBAs, Turnbull’s Blue analogues (TBAs, with the general formula of M_12_^II^[Fe^III^(CN)_6_]_8_ • 48H_2_O, where M is a divalent metal ion),^20^ distinguished by the presence of ferrocyanide vacancies, have demonstrated exceptional rate capabilities in acidic electrolytes through rapid proton intercalation and deintercalation. Due to their small size and low mass, protons offer superior kinetic properties as charge carriers, enabling faster diffusion compared to metal ions.^21,22^ Ji et al.^23^ reported that copper-containing TBAs could deliver a capacity of 48 mAh g^–1^ at an impressive charge–discharge rate of 5000 C (where 1 C corresponds to a full charge or discharge in 1 h), retaining 60% of the initial capacity even after 730,000 charge–discharge cycles at 500 C. Such a high rate capability of the copper-containing TBA was recently exploited as the RR material to demonstrate highly rate-mismatched electrochemical synthesis.^2^ In a subsequent study,^24^ nickel-containing TBAs achieved charge–discharge rates up to 6000 C (390 A g^–1^) at room temperature, while maintaining significant capacity even at −40 °C. These results highlight the superior proton transport performance of TBAs in acidic media and underscore the importance of metal selection in tuning rate capability. The choice of metal centers (M = Cu, Ni, Co, Fe, Mn, etc.) may modulate their electrochemical behaviors related to proton transport, such as the redox potential and ion-transport kinetics. To maximize the effectiveness of TBAs, however, a comprehensive understanding of how their metal composition impacts proton transport activity and material stability under experimental conditions remains essential.

The high proton mobility observed in TBAs is primarily attributed to the Grotthuss mechanism,^23,25^ which involves proton conduction through a cooperative network of O–H bonds. The efficiency of this transport process can be enhanced by increasing the concentration of ferrocyanide vacancies^23^ or by elevating the operating temperature.^26^ In addition to the Grotthuss mechanism, the magnetic properties of metal species incorporated into TBAs can also affect proton mobility within the water network. For instance, Ohkoshi et al.^27^ observed interference between magnetic ordering and proton conduction in V_2_[Cr­(CN)_6_]_3_, which was attributed to distortions in the hydrogen-bonding network. Similarly, Krah et al.^28^ employed wave function-based Difference Dedicated Configuration Interaction (DDCI) calculations to demonstrate that the transition of NiFe-PBA from ferromagnetic to antiferromagnetic behavior was associated with structural deformation of the Ni–Fe cyanide bridge, which in turn affected the mobility of Na^+^ ions. Although direct evidence of spin-state-coupled proton transport in PBAs is lacking, cobalt-based spin-crossover (SCO) complexes have shown that spin transitions can influence proton conductivity by reorganizing hydrogen-bond networks.^29^ A recent study on a Hofmann-type metal–organic framework (MOF) further demonstrates that spin-state switching can couple with redox activity and electron transfer.^30^ Despite these insights, most theoretical studies neglect explicit spin-flipping during ion transport.^31^

In addition to ionic conductivity, the stability of the electrode material during ion intercalation and deintercalation is a critical performance metric for RRs. In the broader class of PBAs (including TBAs), the dissolution of transition metal ions from TBAs can significantly affect their stability in electrolytes.^32,33^ In PBAs, transition metal ions are known to leach from the solid framework and migrate into the electrolyte, leading to gradual cathode degradation.^34^ This dissolution process is further accelerated by the presence of water, which enhances the solubility of metal ions.^35^ The effect is particularly pronounced in PBAs containing Jahn–Teller active transition metal ions—such as high-spin Mn^3+^, high-spin Fe^4+^, and low-spin Ni^3+^—where a single electron occupies two degenerate e_g_ orbitals of the six-coordinated transition metal ion.^36,37^ The Jahn–Teller effect, arising from the presence of partially filled d-orbitals, induces lattice distortions that lift the degeneracy of these orbitals and reduce structural stability. As a result, PBAs containing such species exhibit increased susceptibility to degradation under acidic conditions.^38^ Thus, the choice of metal ions in TBAs critically influences not only proton transport activity but also dissolution behavior, ultimately impacting the overall material stability.

Despite these advances, a systematic investigation into the proton transport activity and stability of TBAs synthesized with different metal ions remains limited. In this study, we combined theoretical and experimental approaches to evaluate how the incorporation of various metals (Cu, Zn, Mn, Fe, Co, and Ni) and their associated spin-state dynamics impacts the behavior of TBAs in acidic environments, with a particular focus on proton transport activity and material stability. Density functional theory (DFT) calculations were employed to analyze the energetics of proton transport between water molecules, providing a theoretical framework for assessing proton transport performance. These computational results were compared with the experimentally measured rate capabilities of as-synthesized TBAs in acidic solutions. Additionally, the stability of TBAs with different metal compositions was evaluated by calculating the thermochemistry of metal dissolution under experimental conditions and by monitoring capacity retention over charge–discharge cycling. This integrated approach enabled the establishment of direct correlations between metal identity, proton transport properties, and material stability, providing crucial insights for the rational design of this specific type of high-performance energy storage and conversion materials.

## 2 Results and Discussion

### 2.1 Proton Transport Activity

A comprehensive analysis of proton transport activity in TBAs containing different metal ions was conducted using both first-principles calculations and experimental measurements. DFT calculations were performed to compare the reaction energies associated with proton transport in TBAs incorporating either diamagnetic, paramagnetic, or ferromagnetic metals. This comparison was used to explore how variations in the atomic spin magnetic moments, particularly the incorporation of paramagnetic or ferromagnetic metals with a greater number of unpaired electrons, may influence proton mobility within the structures. In addition, we assessed the energy cost for proton transport without accounting for changes in electron spin orientation across all systems, to examine the potential effect of the inclusion of ferromagnetic metals on the reaction energy. Experimental rate capacity measurements were also conducted to evaluate differences in performance among TBAs synthesized with diamagnetic, paramagnetic, and ferromagnetic metal salts.

#### 2.1.1 Theoretical Calculations

DFT calculations were performed to determine the reaction energies associated with proton transport within the TBA material M_12_^II^[Fe^III^(CN)_6_]_8_·48H_2_O, hereafter referred to as M-TBA, to elucidate the energetics of proton diffusion. While M typically represents a divalent transition metal ion, the post-transition metal Zn^2+^ was also studied. The structural model used in this work was based on the experimentally validated CuFe–PBA framework reported by Wu et al.^23^ In that study, the structure was refined using synchrotron XRD (Fm3-m lattice, a = 10.125 Å) and neutron diffraction on deuterated samples, which resolved three classes of water molecules: ligand water, central zeolitic water, and off-center zeolitic water. The refined positions were further confirmed by electron density mapping and DFT relaxation, ensuring a physically realistic hydrogen-bonding network rather than an arbitrary local minimum. Moreover, stochastic modeling of ferricyanide vacancies demonstrated that more than 95% of the pore network is percolating, enabling long-range hydrogen-bond connectivity essential for Grotthuss conduction. In our calculations, the optimized lattice constants varied only slightly across different metal substitutions (10.15 Å for Cu, 10.19 Å for Zn, 10.26 Å for Fe/Co/Ni, and 10.34 Å for Mn), suggesting that the underlying water network remains essentially unchanged. This experimentally benchmarked and structurally consistent model provides a reliable basis for comparing the effect of different metal species on proton migration. To further confirm its robustness under ambient conditions, we also performed *ab initio* molecular dynamics (AIMD) simulations for Cu-TBA at 300 K for a 5 ps production run following equilibration. Although this sampling length is moderate, it is consistent with previous AIMD studies on hydrated and confined-water systems,^39–41^ where comparable trajectories were sufficient to assess structural stability once equilibrium was reached. These simulations showed that the 0 K DFT geometry remains dynamically stable with only modest thermal broadening of the water network (see SI, Section S4). The radial distribution functions (RDFs) of O–O, O–H, and H–H pairs retain their first-shell peaks near the corresponding 0 K DFT values, with thermal broadening at 300 K. The root-mean-square deviation (RMSD) of water oxygen positions, computed in a permutation-invariant manner, fluctuates within ∼1.1–1.65 Å, well below the O···O nearest-neighbor distance (∼2.7 Å), indicating only localized excursions. Complementary oxygen occupancy maps projected along the crystallographic XY, XZ, and YZ planes further confirm that water molecules fluctuate around their equilibrium sites without evidence of migration into alternative metastable positions. Together, these AIMD analyses demonstrate that the confined water network remains dynamically stable at room temperature.

As shown in Figure 1b, the M-TBA structure was constructed by removing four ferrocyanide ions [Fe^III^(CN)_6_]^3–^ (Figure 1c) from every three unit cells of the Berlin green analogue M_4_^III^[Fe^III^(CN)_6_]_4_·8H_2_O (BGA, Figure 1a), consistent with the structure reported in a previous study.^18^ The framework of one TBA unit cell therefore comprises three BGA units. Six water molecules were subsequently introduced into the TBA unit cell, coordinating with six M ions (depicted as blue spheres in Figure 1) adjacent to each ferrocyanide ion vacancy (highlighted by the green dashed rectangle in Figure 1b). Water molecules directly coordinated to M ions are referred to as “*ligand water*”, whereas those within the cages, without direct metal coordination, are termed “*zeolitic water*”.^23^ The coordination between ligand water molecules and M ions suggests that altering the M species could affect proton transport activity within the M-TBA material.

**Figure 1:**
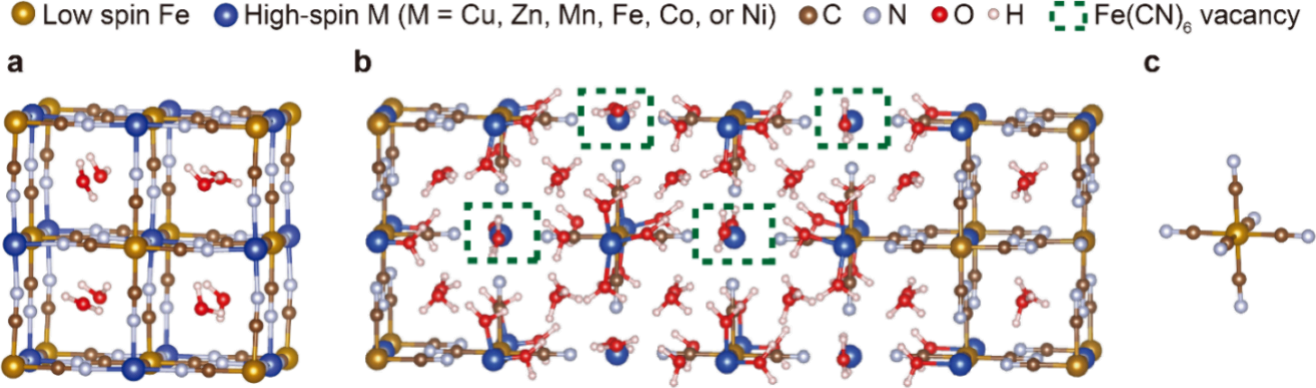
Crystal structures of (a) Berlin Green analogue (BGA) M_4_^III^[Fe^III^(CN)_6_]_4_·8H_2_O, (b) Turnbull’s Blue analogue (TBA) M_12_^II^[Fe^III^(CN)_6_]_8_·48H_2_O, and (c) molecular structure of ferrocynide ion [Fe^III^(CN)_6_]^3–^.

To explore this effect, we systematically investigated the impact of the identity of the M ion on the reaction energies associated with proton transport. The selected M ions included diamagnetic metals with nearly fully paired electrons (Cu and Zn), as well as paramagnetic and ferromagnetic metals with unpaired electrons (Mn, Fe, Co, and Ni). As illustrated in Figures 2a and 3a, the proton transport process analyzed corresponds to the diffusion of the hydrogen atom labeled in green (“Diffusing H”) within a hydronium ion, moving from a zeolitic water molecule located in a high-density water region within a BGA unit (initial state, IS) to a zeolitic water molecule positioned at the boundary between two adjacent BGA units (final state, FS). The most stable proton binding configurations, either to ligand water or zeolitic water molecules, were selected as the IS and FS, respectively.

**Figure 2:**
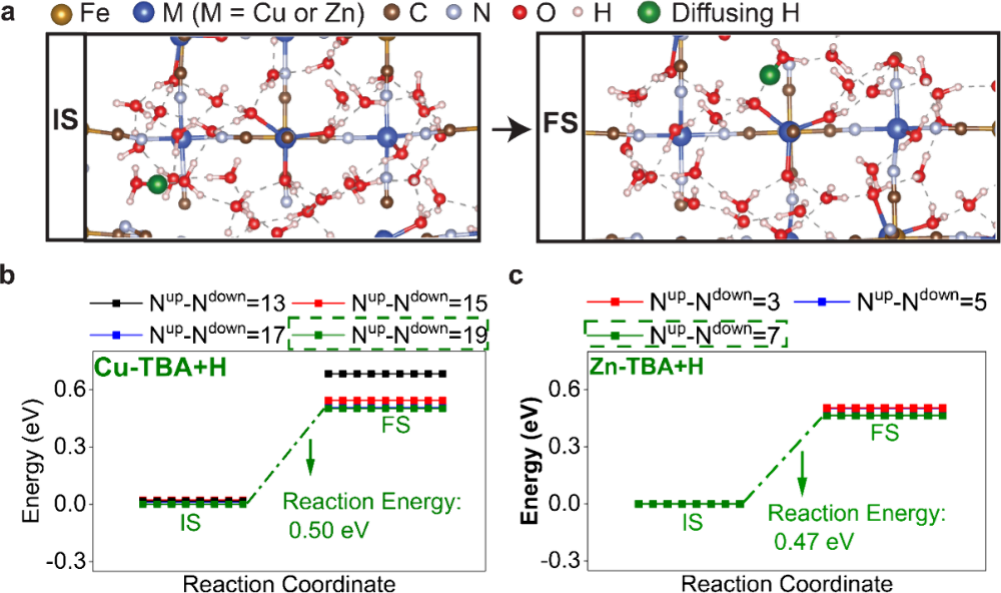
(a) Minimum energy structures illustrating the initial (IS) and final states (FS) for proton transport from a zeolitic water molecule within a Berlin Green analogue (BGA) unit to another water molecule at the boundary between two neighboring BGA units in the M-TBA+H (M = Cu, Zn). Energy profiles of this process are shown for Cu- (b) and Zn- (c) TBA+H. “N^up^-N^down^” denotes the difference in electron numbers between spin-up and spin-down states, with the most energetically favorable value highlighted by a dashed rectangle in the N^up^-N^down^ legend. The green lines represent states where no electron flipping occurs in high-spin and low-spin metal ions coordinated to N and C, respectively. The remaining colored lines correspond to the states resulting from electron flips occurring in these green line states.

**Figure 3:**
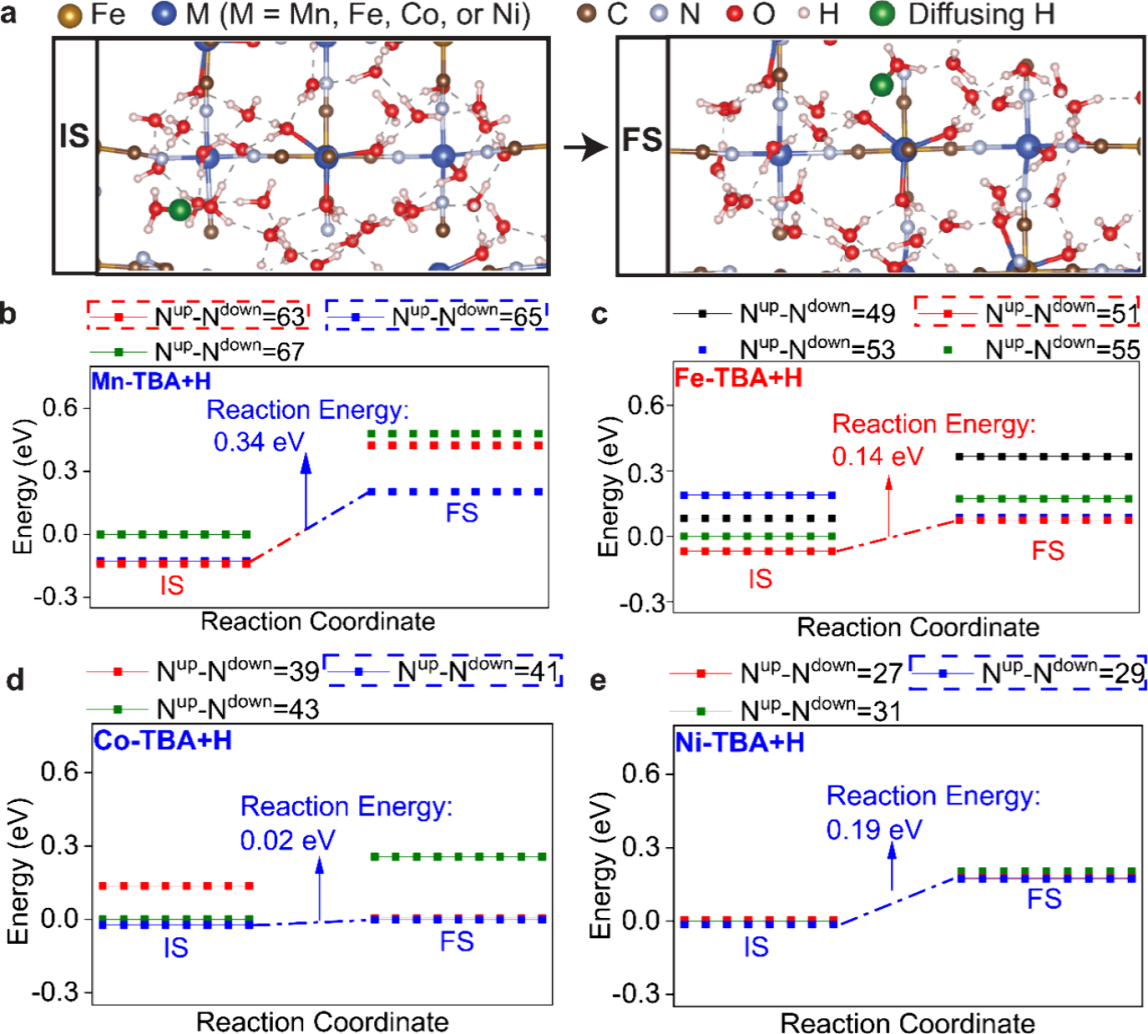
(a) Minimum energy structures illustrating the initial (IS) and final states (FS) for proton transport from a zeolitic water molecule within a Berlin Green analogue (BGA) unit to another water molecule at the boundary between two neighboring BGA units in the M-TBA+H (M = Mn, Fe, Co, or Ni). Energy profiles of this process are shown for Mn- (b), Fe- (c), Co- (d), and Ni- (e) TBA+H. “N^up^-N^down^” denotes the difference in electron numbers between spin-up and spin-down states, with the most energetically favorable value highlighted by a dashed rectangle in the N^up^-N^down^ legend. The green lines represent states where no electron flipping occurs in high-spin and low-spin metal ions coordinated to N and C, respectively. The remaining colored lines correspond to the states resulting from electron flips occurring in these green line states.

Figures 2 and 3 present the calculated energetics of proton transport across different M-TBAs. Figure 2 summarizes the results for Cu- (Panel b) and Zn-TBA (Panel c). Figure 3 shows those for Mn- (Panel b), Fe- (Panel c), Co- (Panel d), and Ni- (Panel e) TBA. In these calculations, different spin states were considered by varying the difference between spin-up and spin-down electrons (N^up^-N^down^) to account for the magnetic properties of the system. The energetics corresponding to high-spin M ions and low-spin Fe ions coordinated to N and C atoms, respectively,^42,43^ are depicted by green lines in Figures 2 and 3. Low-spin Fe ions were selected based on the strong ligand field exerted by the carbon end of the cyanide ligand, which favors low-spin configurations in both Fe^2+^ and Fe^3+^ under typical TBA coordination environments. To further explore the influence of electron spin-flipping, spin orientation reversals at low-spin Fe ions were introduced, generating additional spin states represented by blue, red, and black lines. Electron spin-flipping on metal ions coordinated to nitrogen was excluded, as these configurations were consistently higher in energy compared to those involving Fe ions coordinated to carbon. Spin-flipping was introduced progressively across more Fe sites until the energies of both the IS and FS either increased or remained unchanged, indicating the stabilization limit. The IS without spin-flipping (green line for IS) was taken as the reference. This comprehensive analysis provides the foundation for understanding how spin states and metal ions collectively influence proton transport energetics within the M-TBA framework.

##### 2.1.1.1 Energetics of Proton Transport in Diamagnetic M-TBAs

In TBAs containing diamagnetic metals, such as Zn- and Cu-TBA, the d orbitals of Zn^2+^ ions are fully occupied with paired electrons, while Cu^2+^ ions contain a single unpaired electron. Despite this, Cu^2+^ exhibits only weak paramagnetism and is often treated as effectively diamagnetic. Meanwhile, the Fe^3+^ ions within the framework pair their d-orbital electrons as much as possible, resulting in one unpaired electron per Fe^3+^ site. Upon proton intercalation, one Fe^3+^ ion undergoes reduction, as reflected by a decrease in its atomic charge from around +0.23e to +0.13e evaluated by a charge partitioning scheme.^44,45^ Consequently, the H-intercalated Cu-TBA (Cu-TBA+H) and Zn-TBA (Zn-TBA+H) systems contain 19 and 7 unpaired electrons per unit cell, respectively, for the green line spin states shown in Figure 2. In these systems, the electron spin configurations remain unchanged during proton transport, as indicated by the lower energies of the green lines compared to other spin-flipped states (blue, red, and black lines). The reaction energies for proton transport in Zn-TBA+H and Cu-TBA+H are calculated to be 0.47 and 0.50 eV, respectively. These comparable values indicate that the incorporation of different diamagnetic metals might have minimal impact on the proton transport energetics for this class of M-TBAs.

##### 2.1.1.2 Energetics of Proton Transport in Paramagnetic and Ferromagnetic M-TBAs

In contrast, substituting the M ions with a paramagnetic metal (Mn) or ferromagnetic metals (Fe, Co, and Ni) alters the ground states associated with proton transport (Figure 3). In these systems, the lowest energy states for both the IS and FS shift from the green-line configurations observed in Cu- and Zn-TBA+H (Figure 2) to the blue or red-line configurations in the M-TBAs (Figure 3). This shift indicates that one or two instances of electron spin-flipping occur because of paramagnetic or ferromagnetic metals incorporation in the TBA. The introduction of these spin-flipped states leads to a reduction in the reaction energy required for proton transport, particularly in Mn-, Fe-, and Co-TBA+H.

As shown in Figure 3, the high-spin M ions and low-spin Fe ions in M-TBAs containing one H atom per unit cell (M-TBA+H) result in a total of 67, 55, 43, and 31 unpaired electrons for Mn- (Panel b), Fe- (Panel c), Co- (Panel d), and Ni-TBA+H (Panel e), respectively. For Mn-TBA+H, energy analysis reveals that two instances of electron spin-flipping are favored in the IS and one in the FS, reducing the reaction energy from 0.48 eV (green lines) to 0.34 eV (red and blue lines for the IS and FS, respectively). For Fe-TBA+H, the lowest energy configurations, depicted by red lines in both the IS and FS, correspond to two electron spin-flips at low-spin Fe sites, decreasing the reaction energy from 0.17 eV (green lines) to 0.14 eV (red lines). For Co-TBA+H, a more pronounced energy decrease is observed, with the reaction energy decreasing from 0.26 eV (green lines) to 0.02 eV (blue lines) following a single electron spin-flip in both the IS and FS. Similarly, for Ni-TBA+H, the most stable spin states for the IS and FS are identified by the blue lines, corresponding to configurations with one electron spin-flip. However, in this case, the impact on reaction energy is minimal, with a decrease from 0.19 eV by less than 0.01 eV. The energy differences between the different spin states in both the IS and FS remain within 0.02 eV, likely due to the nearly complete filling of the d orbitals with eight electrons per Ni^2+^ ion.

Overall, as summarized in Table 1, the incorporation of paramagnetic or ferromagnetic metals as M ions leads to lower reaction energies for proton transport compared to M-TBAs containing diamagnetic metals. In particular, the nearly thermoneutral proton transport observed in Co-TBA suggests that it can have exceptionally high proton transport activity, facilitated by electron spin-flipping. These results highlight the promoting effect of electron spin-flipping on proton mobility when metals with a greater number of unpaired electrons are incorporated into the TBA framework.

**Table 1:** Calculated Reaction Energy (Δ*E*) for Proton Transport in M-TBA+H (M = Cu, Zn, Mn, Fe, Co, or Ni), based on the Lowest-Energy Spin Configurations Shown in Figures 2a and 3a^a^

Reaction Energy	Cu-TBA+H	Zn-TBA+H	Mn-TBA+H	Fe-TBA+H	Co-TBA+H	Ni-TBA+H
Δ*E* (eV)	0.50	0.47	0.34	0.14	0.02	0.19

a Positive entries reflect endothermic proton transport.

##### 2.1.1.3 Influence of Electron Spin-Flipping on Proton Transport

As discussed above, electron spin-flipping can occur at low-spin Fe ions coordinated to carbon atoms when paramagnetic or ferromagnetic metals are used as M ions. To further elucidate the differences in electron spin state changes during the proton transport process between diamagnetic and magnetic M-TBAs, we analyzed the atomic spin magnetic moments of eight low-spin Fe atoms involved in proton diffusion. These Fe sites are labeled in the IS and FS, corresponding to the diffusion pathway of the green-circled hydrogen atom shown in Figure 4a. These magnetic moments are represented by red bars for the IS and blue bars for the FS. The detailed results for Cu-, Zn-, Mn-, Fe-, Co-, and Ni-TBA+H are shown in Panels b, c, d, e, f, and g of Figure 4, respectively.

**Figure 4:**
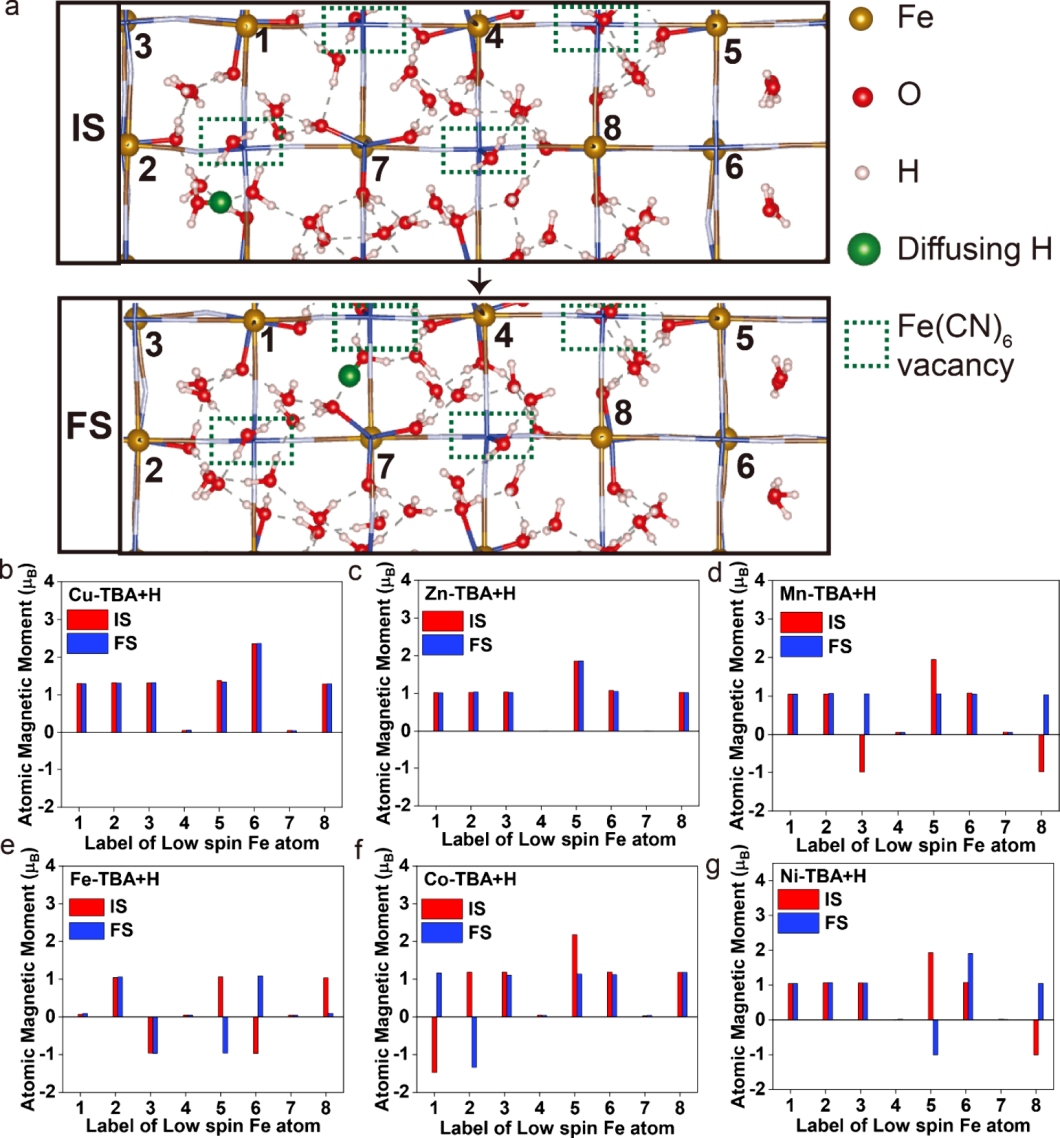
(a) Minimum energy structures of the initial (IS) and final states (FS) for proton transport from a zeolitic water molecule within a Berlin Green analogue unit to another water molecule at the boundary between two neighboring units in M-TBA+H (M = Cu, Zn, Mn, Fe, Co, or Ni). Numerical labels indicate the respective low-spin Fe atoms. Note: to clarify the labeling of Fe atoms, only the Fe, H, and O atoms are shown. (b–g) Atomic spin magnetic moments for the low-spin Fe atoms labeled in panel (a) in both the IS (red bar) and FS (blue bar) of the proton transport process depicted in panel (a) for Cu- (b), Zn- (c), Mn- (d), Fe- (e), Co- (f), and Ni- (g) TBA+H.

Panels b and c illustrate that the magnetic moments of the low-spin Fe atoms remain nearly unchanged during the proton transport process in Cu- and Zn-TBA+H, as indicated by the similar heights of the red and blue bars for each Fe ion. In contrast, in M-TBAs+H incorporating paramagnetic or ferromagnetic metals (M = Mn, Fe, Co, or Ni), the magnetic moments of the low-spin Fe atoms exhibit significant changes between the IS and FS. In particular, the reversal of magnetic moment signs reflects changes in electron spin orientation following the incorporation of paramagnetic or ferromagnetic metal ions. Notably, even though the N^up^-N^down^ values for the IS and FS remain the same in Fe, Co, and Ni-TBA+H (Figure 3), the electron spin orientations of individual low-spin Fe atoms differ between the IS and FS (Figure 4), indicating that the electron spin-flipping occurs during proton diffusion between water molecules in TBAs containing paramagnetic or ferromagnetic metals.

##### 2.1.1.4 Effect of Fe Reduction Behavior on Proton Transport in Ferromagnetic M-TBAs

In addition to the variations induced by electron spin-flipping, significant differences in the reaction energies for proton transport were observed across all M-TBA+H materials, as summarized in Table 2. These reaction energies correspond to the energy levels of the green-line spin states shown in Figures 2 and 3. Cu-, Mn-, and Zn-TBA+H exhibit similarly higher (endothermic) reaction energies compared to Co-, Ni-, and Fe-TBA+H, suggesting slower proton transport in Cu-, Mn-, and Zn-TBA+H.

**Table 2:** Calculated Reaction Energy (Δ*E*) for Proton Transport without Electron Spin Flipping, Hydrogen Binding Energies in the IS (*E*_ads,H_^IS^) and FS (*E*_ads,H_^FS^), and the Atomic Magnetic Moment Changes (Δ*M)* of Fe Atoms during the H Intercalation Process Forming the IS^a^

Material	Δ*E* (eV)	*E*_ads,H_^IS^ (eV)	*E*_ads,H_^FS^ (eV)	Δ*M*_Fe‑1_ (μ_B_)^a^	Δ*M*_Fe‑3_ (μ_B_)^a^	Δ*M*_Fe‑7_ (μ_B_)^a^
Cu-TBA+H	0.50	–4.24	–3.74	+1.05→+1.06	+1.07→+1.07	–0.39→+0.05
Mn-TBA+H	0.48	–3.72	–3.24	+1.05→+1.05	+1.59→+1.09	+0.44→+0.07
Zn-TBA+H	0.47	–3.94	–3.47	+1.02→+1.02	+1.03→+1.04	+0.98→+0.01
Co-TBA+H	0.26	–3.02	–2.76	+1.05→+1.03	+1.92→+1.06	+0.05→+0.04
Ni-TBA+H	0.19	–2.83	–2.64	+1.05→+1.04	+1.93→+1.06	+0.04→+0.02
Fe-TBA+H	0.17	–3.12	–2.95	+1.04→+0.07	+0.87→+1.07	+0.03→+0.04

a Electron spin flipping shown as green line states in Figures 2 and 3. Note that Δ*E* values in this table are identical to those in Table 1, only for Cu-, Zn-, and Ni-TBA+H.

b Δ*M*_Fe‑1,_ Δ*M*_Fe‑3_, and Δ*M*_Fe‑7_ refer to the change in magnetic moment of the Fe atoms labeled “1” (Fe-1), “3” (Fe-3), and “7” (Fe-7) in Figure 4a, during the H intercalation process to form the IS. Fe-1, Fe-3, and Fe-7 ions are adjacent to two, one, and three ferrocyanide vacancies, respectively, within a single M-TBA unit cell. The reference for calculating *E*_ads,H_^IS^ and *E*_*ads,H*_^FS^ is a hydrogen atom in the gas phase.

To investigate the origin of this difference, we calculated the hydrogen binding energies at the IS (*E*_ads,H_^IS^) and FS (*E*_ads,H_^FS^) for each M-TBA+H material, as listed in Table 2. The results show that both *E*_ads,H_^IS^ and *E*_ads,H_^FS^ are higher in absolute value for Cu-, Mn-, and Zn-TBA+H than for Co-, Ni-, and Fe-TBA+H. Notably, the differences in *E*_ads,H_ are more pronounced at the IS than at the FS. Therefore, we focused our subsequent analysis on understanding the impact of metal ions on the *E*_ads,H_^IS^

Across all M-TBA materials studied here, the reduction of Fe ions during proton intercalation occurs at sites near the diffusing proton, specifically at Fe-1, Fe-3, or Fe-7 (as labeled in Figure 4a), depending on the identity of the M ions. In Cu-, Mn-, and Zn-TBA+H, the Fe-7 ion—positioned adjacent to three ferrocyanide vacancies—undergoes reduction during H intercalation. This reduction is reflected in a decrease in the atomic magnetic moment from a nonzero value to nearly zero (Δ*M*_Fe‑7_ in Table 2) and in a corresponding decrease in atomic charge, from approximately +0.12e (Cu- and Zn-TBA+H) and −0.04e (Mn-TBA+H) to approximately −0.10e, as determined by the charge partitioning scheme we used.^44,45^

In contrast, in Fe-, Co-, and Ni-TBA+H, the Fe ions that undergo reduction are located at either the Fe-1 or Fe-3 sites, which are surrounded by fewer ferrocyanide vacancies (two vacancies for Fe-1 and one vacancy for Fe-3, as shown in Figure 4a). This reduction is reflected in a decrease in the atomic magnetic moment of approximately 1 μ_B_ (Δ*M*_Fe‑1_ and Δ*M*_Fe‑3_ in Table 2) and in corresponding charge reductions: from around +0.13e to −0.10e for Fe-1 in Fe-TBA+H and from approximately +0.35e to +0.12e for Fe-3 in Co- and Ni-TBA+H.

Additionally, changes in the atomic magnetic moment of Fe-3 in Mn-TBA+H and Fe-TBA+H (Δ*M*_Fe‑3_ in Table 2) are attributed to additional reduction and oxidation processes, respectively, during proton intercalation. This behavior is reflected in charge variations: in Mn-TBA+H, the charge on Fe-3 decreases from +0.26e to +0.13e, indicating further reduction; whereas in Fe-TBA+H, the charge increases from +0.05e to +0.11e, indicating oxidation. Notably, the further reduction of Fe-3 in Mn-TBA+H resembles the reduction behavior observed in Co- and Ni-TBA+H, which likely contributes to the lower reaction energy for proton transport in Mn-TBA+H compared to Cu-TBA+H and Zn-TBA+H.

Overall, the incorporation of ferromagnetic metals (Fe, Co, and Ni) promotes the reduction of Fe ions located farther from dense ferrocyanide vacancies during H intercalation. This destabilizes the most stable IS configuration, where H binds to water in a high-density environment, and lowers the reaction energy required for proton transport.

To evaluate possible structural reorganization associated with proton transfer, we analyzed bond-length statistics within the local coordination environments of the Fe and M centers. Across all M-substituted TBAs studied, the average Fe–C bond length remains unchanged between the optimized IS and FS geometries. The corresponding variations in the average M-N bond lengths are also minimal, within 0.01 Å (Table S2). In addition, bond-length spreads (δ*d* = *d*_max_ - *d*_min_), used to quantify local coordination distortions, exhibit only small variations between IS and FS (Table S3). These results indicate that proton transport induces only minor framework distortion and limited structural reorganization. Accordingly, Jahn–Teller-related structural reorganization is considered to be negligible under the conditions modeled.

#### 2.1.2 Experimental Measurements and Analysis

Building on our theoretical predictions on proton transport in TBAs, we synthesized M-HCF (M = Cu, Zn, Mn, Fe, Co, and Ni) materials using a coprecipitation method and experimentally evaluated their electrochemical behavior. The morphology and composition of the materials were characterized following procedures similar to those reported previously,^2,5,10^ to validate the computed trends. Although commonly referred to as hexacyanoferrates (HCFs) in the literature, our structural and compositional analyses confirmed a M:Fe stoichiometry consistent with the TBA framework employed in our theoretical study, therefore, herein we will use the names MHCF and M-TBA interchangeably. Proton mobility within these materials was experimentally evaluated by measuring their electrochemical performance at various charge–discharge rates (C-rates), up to 100 C. A C-rate of *n* corresponds to the current required to fully charge/discharge the electrode within 1/*n* hours.

We first examined the morphology and composition of the as-synthesized M-HCF materials. Scanning electron microscopy (SEM) images revealed nanosized particles, approximately 20–50 nm in size, for CuHCF, FeHCF, CoHCF, and NiHCF (an example of Co-HCF shown in Figure 5a and all images shown in Figure S1). In contrast, ZnHCF particles are significantly larger, ranging from 200 to 400 nm (Figure S1b) and MnHCF forms cubic structures with lengths of approximately 800 nm (Figure S1c). Powder X-ray diffraction patterns of various as-synthesized M-HCF materials display the characteristic (200), (220), and (400) diffraction peaks, which match well with the standard pattern of the cubic PBA phase (JCPDS number 52–1907) (Figure S2). Furthermore, inductively coupled plasma optical emission spectroscopy (ICP-OES) and thermogravimetric analysis (TGA) were employed to determine the chemical compositions. As summarized in Table S1, the measured M:Fe ratios for all samples were approximately 1.5, in good alignment with the theoretical ratio for TBAs. These results confirm that the synthesized M-HCF materials are structurally and compositionally consistent with the intended M-TBAs.

**Figure 5:**
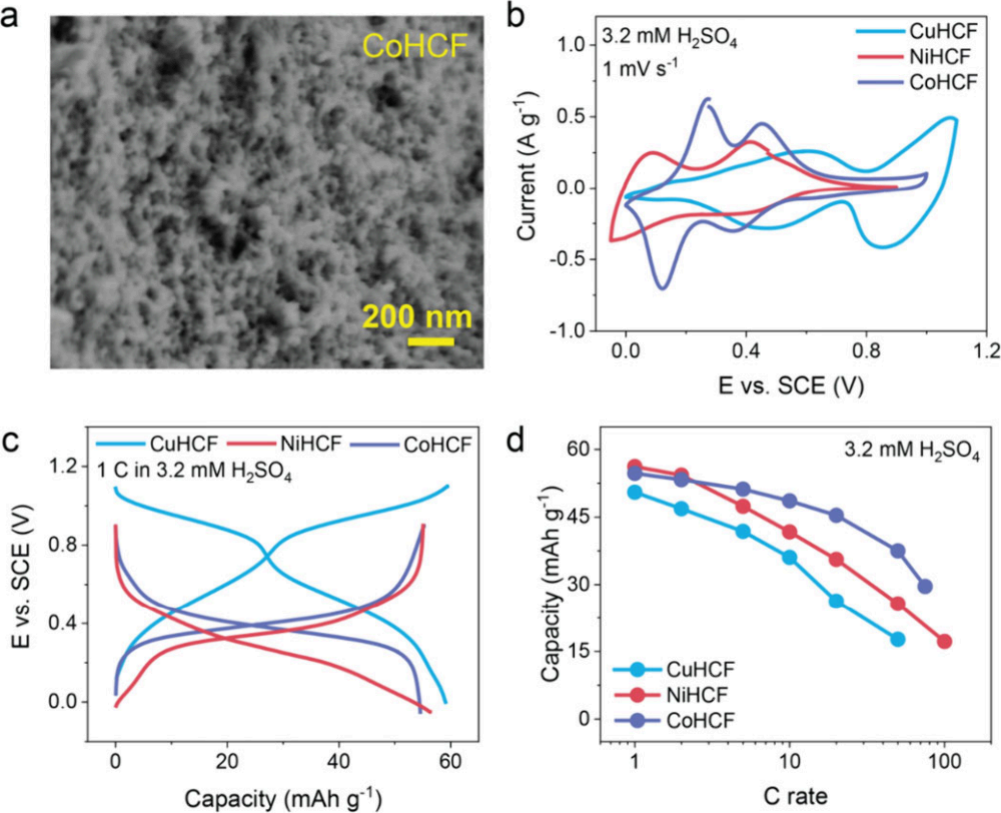
Electrochemical characterizations on typical M-HCF electrodes. (a) SEM image of the as-synthesized CoHCF nanoparticles. (b) Cyclic voltammograms of the M-HCF (M = Cu, Ni, and Co) electrodes at 1 mV s^–1^ in 3.2 mM H_2_SO_4_ solution. (c) Galvanostatic charge–discharge profiles of the M-HCF (M = Cu, Ni, and Co) electrodes at 1 C in 3.2 mM H_2_SO_4_ solution. Here, 1 C rate is 65 mA g^–1^ based on their theoretical capacity. (d) Rate capability of the M-HCF (M = Cu, Ni, and Co) electrodes in 3.2 mM H_2_SO_4_ solution. For (b), (c), and (d), NiHCF and CoHCF electrodes were evaluated in 3.2 mM H_2_SO_4_ solution with 50 mM NiSO_4_ or CoSO_4_, respectively, to suppress their dissolution.

We then evaluated the electrochemical performance of the as-synthesized M-HCF materials in a 3.2 mM H_2_SO_4_ solution. To suppress potential material dissolution, 50 mM of Zn^2+^, Mn^2+^, Fe^2+^, Co^2+^, and Ni^2+^ salts were added to their respective electrolytes. However, due to the competitive Zn^2+^ intercalation in ZnHCF (Note S2 and Figure S3) and the instability of FeHCF and MnHCF in the electrolyte (Figures S4–S9), our study primarily focused on the stable CuHCF, NiHCF, and CoHCF materials, as shown in Figure 5b–d. The higher acid tolerance of CuHCF may be influenced by metal–ligand bond thermodynamics, consistent with the Irving–Williams’ series (Mn < Fe < Co < Ni < Cu > Zn). Stronger Cu–N­(CN) coordination makes the cyanoferrate framework less susceptible to proton-assisted ligand substitution and metal dissolution compared with the Ni^2+^/Co^2+^ analogues, consistent with our computational results in the next section. The cyclic voltammograms in Figure 5b reveal the peaks associated with proton intercalation and release, which typically produce multiple sets of peaks because protons can insert into the lattice under different hydration environments through hydrogen-bonding interactions.^23^ The capacity of CuHCF in concentrated acidic solution (2 M H_2_SO_4_) is higher than that in diluted acidic solution (3.2 mM H_2_SO_4_) due to the higher concentration of protons. At C-rates above 2 C, the capacity of these materials follow the trend: CoHCF > NiHCF > CuHCF, suggesting that CoHCF maintains better performance under moderate-to-high charge–discharge conditions, making it a more favorable candidate for high-power applications.

To interpret these experimental differences in rate performance, we refer to the theoretical proton transport energetics previously calculated for M-TBA+H (Figure 6a). As discussed earlier, a lower reaction energy corresponds to more favorable proton mobility. The computed trend—Co-TBA+H > Fe-TBA+H > Ni-TBA+H > Mn-TBA+H > Zn-TBA+H > Cu-TBA+H—predicts enhanced transport activity in Co- and Ni-based materials, in good agreement with the observed discharge capacities.

**Figure 6:**
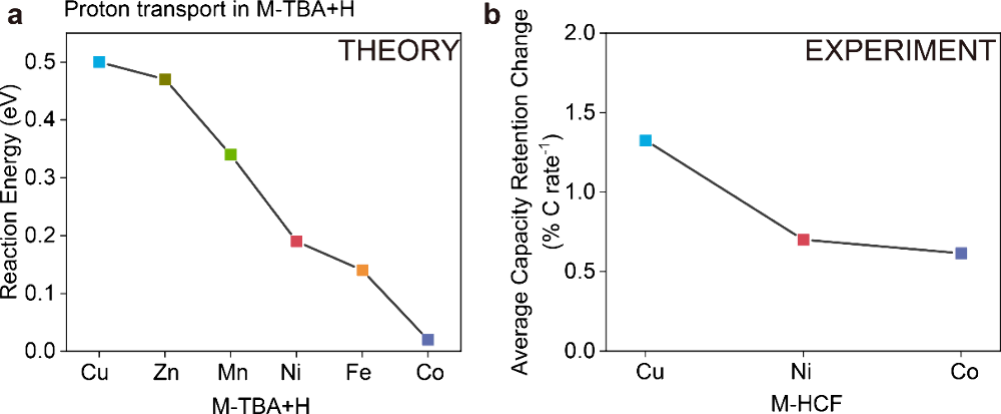
(a) Reaction energy calculated for proton transport from a zeolitic water molecule within a Berlin Green analogue (BGA) unit to another one at the boundary between two neighboring BGA units in M-TBA+H (M = Cu, Zn, Mn, Fe, Co, or Ni). (b) Experimentally determined average capacity retention change of M-HCF (M = Cu, Ni, or Co), defined as the average decrease of the capacity retention (in units of %) of the M-HCF electrode as the C-rate increases by 1 C, based on the capacity changes with varying C-rates up to 100 C shown in Figure 5d. Here, 1 C is 65 mA g^–1^ based on their theoretical capacity. Note: Lines are drawn as visual guides to highlight trends and do not represent linear fits.

We further quantified rate performance by calculating the average capacity retention changes with C rate increase of these TBAs based on their rate capabilities (Figure 5d). Summarized in Figure 6b, this metric reflects the capacity loss from 1 C to the highest tested C-rate, with a steeper decline indicating poorer rate performance. Among these three materials, CuHCF demonstrates the highest capacity retention loss, approximately 1.33% per 1 C increase, reflecting its inferior rate performance compared to NiHCF (0.70%) and CoHCF (0.62%), with CoHCF showing the best performance. Microstructural factors and electronic transport may also influence apparent rate capability. Since the sizes and morphologies of these MHCFs are similar (Figure S1), the differences in rate capability are more likely determined by their compositions. Differences in electronic conductivity/polarization (e.g., *iR* drop/voltage hysteresis) may additionally contribute to their different rate capability. Taken together, the quantitative consistency between the experimental trend in Figure 6b and the theoretical prediction in Figure 6a further supports the connection between intrinsic proton transport properties and rate capability. We note that the correlation in Figure 6 points to an LFER-like correlation between a thermodynamic descriptor (reaction energy) and an experimentally observed kinetic response; thus, it should be interpreted as a heuristic trend rather than a mechanistic proof.

Additionally, it is worth noting that without electron spin-flipping, the reaction energy for proton transport in Co-TBA+H is 0.26 eV (as indicated by green lines in Figure 3d), which is higher than the corresponding value of 0.19 eV for Ni-TBA+H (green lines in Figure 3e). This observation alone, however, cannot fully account for the superior rate performance of CoHCF (shown in Figure 6b). When electron spin-flipping is considered, the reaction energy for Co-TBA+H significantly decreases to 0.02 eV (blue lines in the IS and FS, Figure 3d), aligning with the highest experimental capacity observed for CoHCF. These results suggest that electron spin-flipping plays a critical role in enhancing the proton transport activity in Co-TBA.

### 2.2 Material Dissolution in Acidic Solution

Following the identification of enhanced proton mobility in M-TBAs incorporating ferromagnetic metals as M ions, we evaluated their stability by examining material dissolution in acidic solutions. To assess the dissolution trends, we combined theoretical thermochemical calculations with experimental measurements of capacity retention under electrochemical cycling.

#### 2.2.1 Theoretical Calculations

Transition metal ions in PBAs can dissolve into solution as salts, and therefore higher concentrations of metal ions in solution can suppress further dissolution.^46^ Consequently, the degradation of TBAs may proceed via the dissolution of both TBA (M_12_[Fe­(CN)_6_]_8_·48H_2_O) and its protonated form, TBA+H (HM_12_[Fe­(CN)_6_]_8_·48H_2_O), as represented by eqs 1 and 2:

1 $$M_{12}{\left[\mathrm{Fe}{\left(\mathrm{CN}\right)}_{6}\right]}_{8}\cdot 48H_{2}O\to 8\mathrm{Fe}{{\left(\mathrm{CN}\right)}_{6}}^{3-}+12M^{2+}+48H_{2}O$$

2 $${\mathrm{HM}}_{12}{\left[\mathrm{Fe}{\left(\mathrm{CN}\right)}_{6}\right]}_{8}\cdot 48H_{2}O\to H^{+}+8\mathrm{Fe}{{\left(\mathrm{CN}\right)}_{6}}^{3-}+12M^{2+}+48H_{2}O+e^{-}$$

The dissolution process described above becomes more pronounced during charging–discharging cycles. Therefore, the dissolution of TBA+H, which is coupled to the electrode potential, is considered the primary contributor to material instability. Additionally, under strongly acidic conditions, Fe­(CN)_6_^3–^ can further dissociate into Fe^3+^ and hydrogen cyanide (HCN), as shown in the eq 3:

3 $$\mathrm{Fe}{{\left(\mathrm{CN}\right)}_{6}}^{3-}+6H^{+}\to {\mathrm{Fe}}^{3+}+6\mathrm{HCN}$$

Thus, the overall dissolution process, combining the reactions described in eqs 2 and 3, is summarized in eq 4:

4 $${\mathrm{HM}}_{12}{\left[\mathrm{Fe}{\left(\mathrm{CN}\right)}_{6}\right]}_{8}\cdot 48H_{2}O+47H^{+}\to 8{\mathrm{Fe}}^{3+}+48\mathrm{HCN}+12M^{2+}+48H_{2}O+e^{-}$$

Accordingly, we calculated the thermochemistry of this dissolution process for M-TBA+H (M = Cu, Zn, Mn, Fe, Co, or Ni) based on eq 4. The chemical potential of M-TBA+H adopted the DFT-calculated electronic energy of the IS of proton transport, as the IS is energetically more stable than the FS (Figures 2 and 3). The chemical potential of the proton in solution, μ­(H^+^), under specific conditions (temperature T, pH, and electrode potential U_SHE_) was determined using the computational hydrogen electrode (CHE) approach, assuming equilibrium with the H_2_ molecule (μ­(H_2_, g)) under the standard condition (1 atm, 298.15 K), as introduced by Nørskov.^47,48^ The equation below illustrates this relationship:

$$\mu \left(H^{+}\right)+\mu \left(e^{-}\right)=0.5\times \mu \left(H_{2},g\right)-eU_{\mathrm{SHE}}-k_{B}T\times \ln10\times \mathrm{pH}$$ 5

Meanwhile, sulfate (SO_4_^2–^) and metal ions (M^2+^) are assumed to be in equilibrium with their corresponding metal sulfate solids MSO_4_ (s). Therefore, the chemical potential of M^2+^ is given by μ­(MSO_4,_ s) - μ­(SO_4_^2–^). Based on these assumptions, the Gibbs free energy change for the dissolution of M-TBA+H can be determined using eq 6 (see more details in Section S3 in the Supporting Information):

$$\Delta G_{M}=\left[12\times \mu \left({{\mathrm{MSO}}_{4}}_{,}s\right)-\mu \left(M-\mathrm{TBA}+H\right)-eU_{\mathrm{SHE}}\right]+8\times \mu \left({\mathrm{Fe}}^{3+}\right)+48\times \mu \left(\mathrm{HCN}\right)-12\times \mu \left({{\mathrm{SO}}_{4}}^{2-}\right)+48\times \mu \left(H_{2}O\right)+0.5\times \mu \left(H_{2},g\right)-k_{B}T\times \ln10\times \mathrm{pH}-48\times \mu \left(H^{+}\right)$$ 6

*U*_SHE_ was taken from experimental redox potentials measured during charge–discharge cycling (Table S1). Under specific experimental conditions, variations in the metal ions (M) affect the term [12 * μ­(MSO_4,_ s) - μ­(M-TBA+H) - e*U*_SHE_] in eq 6. To evaluate the impact of different metal ions on dissolution thermochemistry, we used the Gibbs free energy change of Cu-TBA+H as a reference, thereby eliminating common terms outside the brackets in eq 6, as shown in Figure 7a. The calculated Gibbs free energies of dissolution provide a purely thermodynamic description of the relative stability of the M-substituted TBA frameworks. These values are intended to compare the thermodynamic driving forces for dissolution between materials in this class. Under practical battery operating conditions, degradation processes can also be influenced by kinetic factors, as well as interfacial reactions, surface passivation, and local pH variations, which are not explicitly captured in the present purely thermodynamic analysis.

**Figure 7:**
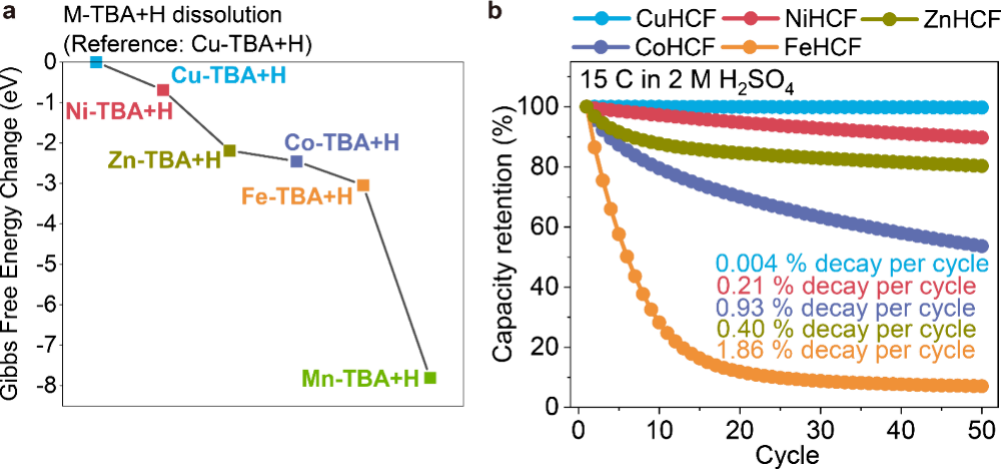
(a) Calculated Gibbs free energy change for the dissolution process of M-TBA+H (M = Cu, Zn, Mn, Fe, Co, or Ni). Line segments between data points are used as guide to the eye. (b) Experimentally measured capacity retention as a function of charge–discharge cycle number for MHCF (M = Cu, Zn, Fe, Co, or Ni) in 2 M H_2_SO_4_ electrolyte at a C-rate of 15 C. Here, 1 C is 65 mA g^–1^ based on their theoretical capacity.

Arrhenius-type relationships were applied to estimate the rate ratios for the dissolution process of M-TBA+H relative to Cu-TBA+H (*r*_M‑TBA+H_/*r*_Cu‑TBA+H_), assuming identical pre-exponential factors for all materials. These rate ratios are used solely as comparative indicators of relative stability. The resulting values, particularly the extremely large ratios obtained for Mn-TBA, should not be interpreted as physically realistic dissolution rates. Instead, they reflect the magnitude of the thermodynamic instability implied by the differences in ΔG. The calculated dissolution rates follow the trend: Mn-TBA+H > Fe-TBA+H > Co-TBA+H > Zn-TBA+H > Ni-TBA+H > Cu-TBA+H (Table 3).

**Table 3:** Values (Unit: eV) Used for Calculating the Theoretical Dissolution Rate Relative to That of Cu-TBA+H (*r*_M‑TBA+H_/*r*_Cu‑TBA+H_) with M = Cu, Zn, Mn, Fe, Co, or Ni, and the Experimental Capacity Retention Decay Per Cycle

Material	12 × μ(MSO_4,_ s) – μ(M-TBA+H)	Δ*G*_M_ – Δ*G*_Cu_	Theoretical *r*_M‑TBA+H_/*r*_Cu‑TBA+H_	Experimental capacity retention decay per cycle
Cu-TBA+H	0.00	0.00	1.00	0.004%
Ni-TBA+H	–0.97	–0.69	4.67 × 10^11^	0.21%
Zn-TBA+H	–1.97	–2.19	1.09 × 10^37^	0.40%
Co-TBA+H	–2.60	–2.45	2.72 × 10^41^	0.93%
Fe-TBA+H	–3.32	–3.04	2.59 × 10^51^	1.86%
Mn-TBA+H	–7.86	–7.81	7.74 × 10^131^	Very unstable^a^

a Note: MnHCF exhibits rapid capacity retention decay, rendering it the most unstable material and challenging to measure accurately. Hence, the experimental capacity retention decay per cycle for this material was classified as “very unstable”.

#### 2.2.2 Experimental Validation of Theoretical Predictions on TBA Stability

To correlate experimental results with theoretical predictions regarding the stability of various M-TBAs in acidic electrolytes, we performed cyclic voltammetry (CV) and galvanostatic charge–discharge (GCD) tests on the synthesized Cu-, Zn-, Mn-, Fe-, Co-, and Ni-HCF materials. CV measurements in a 2 M H_2_SO_4_ solution demonstrate that only the CuHCF electrode maintains electrochemical stability (Figure S6), while the other materials exhibit dissolution tendencies. This trend was further validated through GCD testing at a 15 C rate over 50 charge–discharge cycles in the same electrolyte (Figure S7). Figure 7b shows the capacity retention as a function of charge–discharge cycle number, highlighting the average capacity retention decay per cycle within 50 charge–discharge cycles. Notably, MnHCF exhibits rapid capacity retention decay, rendering it the most unstable material and challenging to test accurately. Accordingly, MnHCF was excluded from Figure 7b, and its “Experimental capacity retention decay per cycle” was classified as “very unstable” in Table 3.

For all characterized materials, the trend in capacity retention decay follows the order: CuHCF < NiHCF < ZnHCF < CoHCF < FeHCF < MnHCF. This experimentally observed stability sequence is consistent with the theoretical rate ratios predicted for the material dissolution process. Importantly, Co- and Ni-TBA, which demonstrate superior proton transport activity compared to Cu-TBA, also exhibit moderate stability against dissolution. In particular, Ni-TBA combines comparable stability to Cu-TBA with enhanced proton transport performance, suggesting its potential for high-rate, durable applications.

Moreover, the dissolution of M-TBAs can be mitigated by adding M^2+^ salts into the electrolyte. This effect is evidenced by the improved rate capacities of NiHCF and CoHCF upon the addition of Ni^2+^ and Co^2+^ ions, respectively, to the 3.2 mM H_2_SO_4_ solution (Figures S8 and S9). These observations are consistent with the theoretical dissolution process outlined in eq 4, wherein the addition of excess M^2+^ ions shifts the dissolution equilibrium toward the solid phase, thereby enhancing the stability of the M-TBA material. In the absence of M^2+^ additives, the CuHCF electrode shows a higher redox potential compared to the CoHCF and NiHCF electrodes, as illustrated by the CVs (Figure 5b) and GCD profiles (Figure 5c). Upon the introduction of Ni^2+^ and Co^2+^ salts in the electrolyte, the rate capacities of NiHCF and CoHCF surpass that of CuHCF (Figure 5d), further confirming the role of M^2+^ additives in suppressing dissolution and improving material stability.

## 3 Conclusions

We utilized both computational and experimental methods to study the proton transport activity and stability of TBAs to investigate how different metal ions in TBAs impact their properties in acidic solutions, thereby facilitating the design of advanced electrode materials. First-principles calculations revealed that M-TBAs incorporating paramagnetic (Mn) or ferromagnetic metals (e.g., Fe, Co, or Ni) coordinated to nitrogen exhibited lower reaction energies for proton transport compared to those incorporating diamagnetic metals (Cu and Zn). This leads to an enhancement in proton transport activity, which can be attributed to changes in the atomic spin magnetic moments during the proton transport process, particularly electron spin-flipping at low-spin Fe sites. Moreover, the incorporation of ferromagnetic metals (Fe, Co, and Ni) further reduces the reaction energy by promoting the reduction of Fe ions located farther from dense ferrocyanide vacancy regions during proton intercalation. This reduction destabilizes the most stable initial state, where the proton is coordinated to water molecules in a densely hydrated environment, thereby facilitating proton transport. These theory-derived trends were confirmed by experimentally measured rate capabilities of as-synthesized M-TBAs. In addition, we assessed material stability by calculating the Gibbs free energy change for the material dissolution process in acidic solutions. The stability trends predicted by our theoretical calculations were consistent with experimental measurements of capacity retention over extended cycling.

Based on this integrated evaluation, Ni-TBA was identified as offering both superior proton transport activity, whereas its stability is comparable to that of Cu-TBA. Importantly, our findings also suggest that spin state modulation through choice of magnetic metal ions or external perturbations could serve as a broader strategy to tune ionic conductivity and catalytic behavior in open-framework materials. Finally, we propose that combining the high proton transport activity of ferromagnetic metals with the enhanced stability associated with diamagnetic metals, through strategies such as doping or core–shell nanostructuring.^19^ Doping can modulate the local electronic environment and redox potential for M-TBAs, while core–shell architectures can physically separate active and protective domains to mitigate structural degradation. These strategies may provide a promising pathway for the development of next-generation, high-performance proton-ion battery electrode materials.

## 4 Methods

### 4.1 Computational

Spin-polarized periodic first-principles calculations were performed using density functional theory (DFT) as implemented in the Vienna ab initio simulation package (VASP).^49,50^ The projected-augmented wave (PAW)^51,52^ method was used in combination with the generalized gradient approximation (GGA) as parametrized by the Perdew–Burke–Ernzerhof (PBE) functional.^53^ A plane wave basis set with a kinetic energy cutoff of 550 eV was employed. To more accurately describe the highly localized d states of metal atoms, the PBE + U approach^54^ was applied, with U – J parameters^42^ of 7.0 or 3.0 eV for metal atoms coordinated to nitrogen or carbon atoms, respectively. The initial magnetic moments of Mn, Fe, Co, and Ni atoms coordinated to nitrogen were set to be 5.0, 5.0, 3.0, and 2.0 μ_B_, respectively. To explore the ground states, various spin configurations were examined by adjusting the difference between spin-up and spin-down electrons (N^up^-N^down^) and assigning initial positive or negative magnetic moments to selected atoms, thereby tuning the spin orientation. Structural optimizations were considered converged when the forces on all atoms were below 0.01 eV/Å. For all open-shell calculations, we used a strict electronic convergence threshold of EDIFF = 1 × 10^–6^ eV, which is more stringent than the value (1 × 10^–5^ eV) typically employed in related PBA/TBA studies.^23,55^ This approach ensured robust SCF convergence and reliable comparison between different spin configurations. A 1 × 1 × 1 Monkhorst–Pack k-point mesh^56^ was used for all structure calculations. The reaction energy (Δ*E*) associated with proton transport was calculated using the following expression: Δ*E* = *E*_FS_ - *E*_IS_, where *E*_IS_ and *E*_FS_ denote the energies of the initial (IS) and final states (FS) of the proton transport step, respectively. The binding energy of hydrogen (*E*_ads,H_) was calculated as *E*_ads,H_ = *E*_TBA‑H_ - *E*_TBA_ - *E*_H_, where *E*_TBA‑H_, *E*_TBA_, and *E*_H_ refer to the total energies of TBA material containing a hydrogen atom per unit cell, the pristine TBA material (with no H in it), and a hydrogen atom (H) in the gas phase, respectively. Dispersion corrections (e.g., PBE+D3 ^57^) were evaluated but found to yield lattice constants that deviated more from experiment when compared to pure PBE. For example, for CuFe-PBA the experimentally measured lattice constant is 10.125 Å^23^, which is in excellent agreement with our non-D3 calculation giving 10.15 Å. In contrast, PBE+D3 predicts a lattice constant of 9.98 Å. On this basis, and consistent with previous PBA/TBA studies by Illas et al.,^58^ and López et al.,^59^ we performed all calculations without D3 corrections.

Proton transfer in PBAs/TBAs is intrinsically coupled to electron spin flipping at Fe sites. Because conventional nudged elastic band (NEB)^60–62^ calculations assume a fixed electronic state along the reaction coordinate, they cannot rigorously capture migration processes involving spin transitions. For this reason, activation barriers were not computed via NEB; instead, relative reaction energies between representative IS and FS were used as a proxy for the spin-proton coupling effect. Δ*E* is employed as a qualitative energetic descriptor to compare relative proton transfer rates across the M-substituted TBA series rather than as a surrogate for activation barriers. This interpretation is consistent with the Brønsted-Evans–Polanyi (BEP) framework, which establishes that activation barriers often scale with reaction energies within families of structurally similar processes. As a precedent, Silaghi et al.^63^ demonstrated a linear correlation between activation energies and reaction energies for analogous bond-breaking processes in zeolite frameworks.

Although a full finite-temperature sampling with ab initio molecular dynamics (AIMD)^64–66^ could in principle provide a more dynamic description of the hydrogen-bond network, this approach is not practical for the present system. As a validation step, we carried out AIMD simulations for Cu-TBA at 300 K (see SI, Section S4), which confirmed that the experimentally benchmarked 0 K DFT structure remains dynamically stable under ambient conditions. However, when an additional proton is introduced, proton transfer in PBAs/TBAs is strongly coupled to the local spin state of Fe sites, and the spin magnetic moments vary with the proton position along the migration path. This would require treating the spin configuration as an additional degree of freedom for each trajectory, which is computationally prohibitive and not feasible within standard AIMD protocols. Accordingly, the AIMD simulations were not intended to probe proton diffusion or to model the proton transport mechanism. Instead, they were designed to assess whether the hydrated framework remains structurally intact over the simulated time scale. The results indicate that no immediate structural collapse or instability occurs under thermal conditions, supporting the mechanical robustness of the framework.

The proposed mechanism involves coupled proton transfer and spin/electronic reconfiguration. Proton hopping via structural diffusion is known to occur on subpicosecond time scales,^67^ while spin crossover/electronic relaxation in Fe-containing systems can occur on femtosecond time scales.^68^ These comparable ultrafast dynamics support the plausibility of a spin-assisted proton-transfer pathway. The present calculations therefore describe relative IS/FS energetics rather than explicit dynamical processes.

### 4.2 Experimental Section

#### 4.2.1 Synthesis of Metal Hexacyanoferrate

Metal hexacyanoferrate (M-HCF, M = Cu, Mn, Fe, Co, or Ni) was synthesized using a coprecipitation method. 40 mL of the corresponding 0.20 M MSO_4_ solution was added dropwise into 40 mL of 0.10 M K_3_Fe­(CN)_6_ solution under vigorous stirring at room temperature. After 6 h of reaction, the precipitate was centrifuged, rinsed with deionized water multiple times, and dried in a vacuum oven at 60 °C overnight. Zinc hexacyanoferrate (ZnHCF) was synthesized using a modified coprecipitation method. Typically, 40 mL of 0.20 M Zn­(NO_3_)_2_ solution was added dropwise into 40 mL of 0.10 M K_3_Fe­(CN)_6_ solution under vigorous stirring at room temperature. After 6 h of reaction, the precipitate was centrifuged, rinsed with deionized water multiple times, and dried in a vacuum oven at room temperature overnight. The phase of ZnHCF was found to be dependent on the drying temperature: the rhombohedral phase was observed when dried at 60 °C and the cubic phase when dried at room temperature.

#### 4.2.2 Materials Characterization

Powder X-ray diffraction (PXRD) patterns of the as-synthesized samples were collected using a Bruker D8 Advance X-ray diffractometer equipped with Cu–Kα radiation. The size and morphology of the samples were characterized using a scanning electron microscope (SEM, Zeiss SUPRA 55VP) equipped with an energy-dispersive X-ray spectroscopy (EDS) detector.

#### 4.2.3 Fabrication and Electrochemical Tests

The M-HCF electrodes were prepared via a conventional slurry-casting method using TUBALL BATT NMP 0.4%, Super P conductive carbon, and the active materials. Typically, 70 wt % active materials and 18 wt % Super P carbon black were ground for 30 min using a pestle and mortar. The mixtures were then added into TUBALL BATT NMP 0.4% that provided 2 wt % SWCNT and 10 wt % polyvinylidene fluoride to form a slurry. One mL NMP was added to the slurry for every 200 mg of active materials to control the viscosity. The slurry was stirred at 700 rpm overnight at room temperature and then cast onto titanium mesh current collectors (150 mesh, with a thickness of ∼ 230 μm). The prepared electrodes were dried in a vacuum oven at 60 °C for 12 h to remove the residual solvent. The areal mass loading was 1 ∼ 3 mg cm^–2^.

The electrochemical performance of the M-HCF (M = Cu, Zn, Mn, Fe, Co, or Ni) electrode was first characterized in 2 M H_2_SO_4_ solution in a three-electrode cell, with a Pt wire counter electrode and a saturated calomel electrode (SCE) as the reference electrode. Cyclic voltammetry (CV) and galvanostatic charge–discharge (GCD) tests of the M-HCF electrodes were recorded on a Bio-Logic VMP-3 multichannel potentiostat. The galvanostatic cycling was performed at a charge–discharge rate of 15 C for stability characterization, where 1 C is defined as 65 mA g^–1^ based on the theoretical capacity of as-synthesized M-HCF materials. The rate capability tests of these M-HCF electrodes were performed in 3.2 mM H_2_SO_4_ solution from 1 to 100 C. CV and rate capability measurements were also performed in 3.2 mM H_2_SO_4_ solution, with the further addition of 50 mM M-salt to aid the stability of ZnHCF, MnHCF, NiHCF and CoHCF electrodes.

## Supplementary Material



## Notes

Experimental details (materials, synthesis, and electrochemical measurements); compositional analysis (ICP-OES); structural characterization (SEM, PXRD); electrochemical characterization (CV, galvanostatic charge–discharge, cycling stability, and rate capability); Zn-ion/proton costorage analysis; theoretical methods and dissolution energetics; AIMD simulations and structural stability analysis; bond-length and coordination analysis; supplementary tables and figures (PDF)
