# Supplementary material for: Trends for Proton Transport Activity and Stability in Turnbull’s Blue Analogues: Theory and Experiments
Source: Chem Mater. 2026 Jun 1;38(12):5903–15. doi: 10.1021/acs.chemmater.5c03404 (PMC13296272; doi:10.1021/acs.chemmater.5c03404)
Supplement: Supplementary file 1 [file cm5c03404_si_001.pdf]

Supporting Information

Trends for Proton Transport Activity and Stability in Turnbull's Blue Analogues: Theory and Experiments

Mengru Li[†], Rui Wang[‡], Pen-Yeh Yen[‡], Anneke Moeller[‡], Reagan Shippy[‡], Katelyn H. Michael[‡], Victor M. Zavala[†], Song Jin[‡*], Manos Mavrikakis[†*]

[†] Department of Chemical and Biological Engineering, University of Wisconsin–Madison, Madison, WI 53706, USA.

[‡] Department of Chemistry, University of Wisconsin–Madison, Madison, WI 53706, USA.

*Corresponding authors:

Song Jin (jin@chem.wisc.edu); Manos Mavrikakis (emavrikakis@wisc.edu)

Note S1. Chemicals and Materials

All chemicals were used as purchased without further purification. Carbon black (Super P Conductive, 99.0+%) was purchased from Alfa Aesar. TUBALL BATT NMP 0.4% (a mixture of single-wall carbon nanotubes, 0.4 wt%; polyvinylidene fluoride, 2 wt%; N-methyl-2-pyrrolidone, > 96.7 wt%) was purchased from OCSiAl. Other chemicals were purchased from Sigma Aldrich. Titanium mesh (150 mesh, with a thickness of ~ 230 μm) was purchased from HeBei ChaoChuang Metal Mesh Co., Ltd (available through Alibaba.com). Deionized nanopure water (18.2 MΩ·cm) from ThermoScientific Barnstead water purification systems was used for all experiments.

Table S1. Weight percent of metal elements and water content in M-TBAs (M = Cu, Zn, Mn, Fe, Co, and Ni) samples determined by ICP-OES measurements.

Material	K wt%	M wt%	Fe wt%	H_2O wt%	M/Fe ratio	Chemical formula
CuHCF	0.60	23.60	13.60	24.30	1.5	$K_{0.06}Cu_{1.5}[Fe(CN)_6] \cdot 5.5H_2O$
ZnHCF	0.96	18.80	11.50	24.40	1.4	$K_{0.12}Zn_{1.4}[Fe(CN)_6] \cdot 6.6H_2O$
MnHCF	2.46	20.20	12.10	23.00	1.7	$K_{0.3}Mn_{1.7}[Fe(CN)_6] \cdot 5.9H_2O$
FeHCF [a]	--	/	41.20	12.70	/	$Fe_{1.5}[Fe(CN)_6] \cdot 2.4H_2O$
CoHCF	1.37	18.50	13.50	24.60	1.3	$K_{0.14}Co_{1.3}[Fe(CN)_6] \cdot 5.7H_2O$
NiHCF	0.90	20.70	13.10	28.60	1.5	$K_{0.1}Ni_{1.5}[Fe(CN)_6] \cdot 6.8H_2O$

Note : [a] The chemical formula of FeHCF is assumed with a $[Fe^{2+}]/[Fe(CN)_6]^{3-}$ molar ratio of 1.5 : 1 based on charge balance.

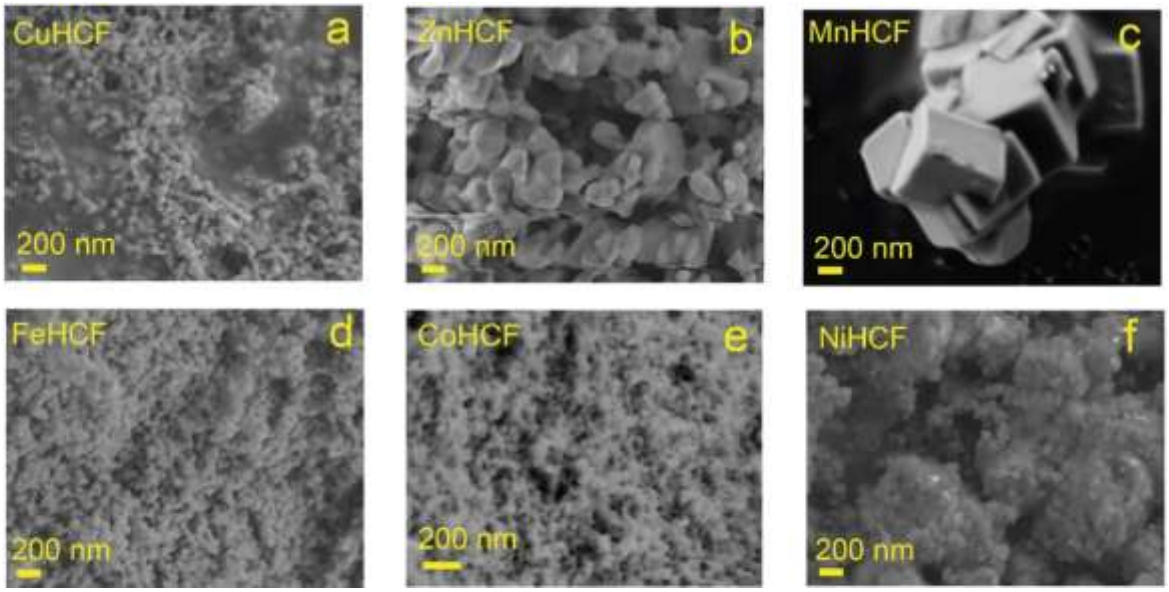

Figure S1. Scanning electron microscopy (SEM) images of as-synthesized M-TBAs. (a) CuHCF. (b) ZnHCF. (c) MnHCF. (d) FeHCF. (e) CoHCF. (f) NiHCF.

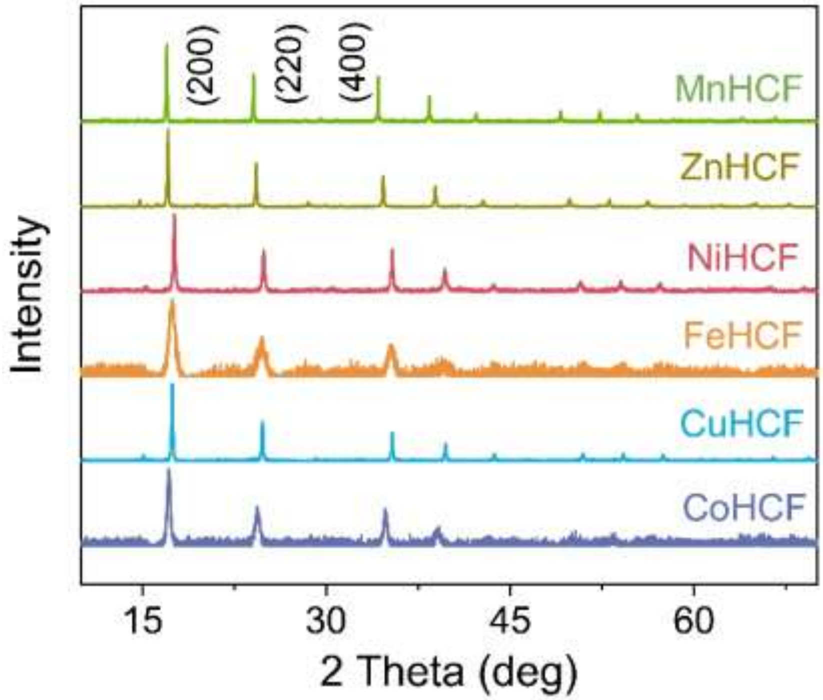

Figure S2. Powder X-ray diffraction (PXRD) patterns of various as-synthesized M-TBAs. The characteristic (200), (220), and (400) diffraction peaks of these samples (MnHCF, ZnHCF, NiHCF, FeHCF, CuHCF, and CoHCF) match well with the standard pattern of the cubic phase of Prussian blue (JCPDS No. 52-1907).

Note S2. Convolution of Zn-ion and proton storage in the ZnHCF material.

The ZnHCF material has been reported for zinc-ion storage in neutral solutions.[1] However, the possible preference for Zn-ion or proton storage in weakly acidic solutions with zinc ions remains unclear. We could study proton transport kinetics if the ZnHCF electrode only stores protons in weakly acidic solutions. Otherwise, the proton transport could not be studied due to the co-storage of proton and zinc ions. Here, we first conducted cyclic voltammograms (CVs) of the ZnHCF electrode in 3.2 mM H_2SO_4 solution and found obvious capacity decay during four cycles, in which the redox potential was about 0.9 V vs. SCE (based on $E_{1/2}$) (Figure S3a). Then, we added 50 mM Zn^{2+} to suppress its dissolution in 3.2 mM H_2SO_4 solution. The CV profiles of the ZnHCF electrode showed increased area and new pairs of redox peaks of ~0.65 V vs. SCE (Figure S3b), indicating that the improved capacity might result from other redox processes rather than proton intercalation. Galvanostatic charge-discharge profiles of the ZnHCF electrode in 100 mM $ZnSO_4$ solution showed obvious Zn intercalation with lower plateaus compared to those in 3.2 mM H_2SO_4 solution with 50 mM Zn^{2+} (Figure S3c). The plateaus of Zn-ion intercalation with lower redox potentials confirmed that these peaks of ~0.65 V vs. SCE were from the zinc-ion storage. Therefore, proton transport in ZnHCF is difficult to study in a conclusive manner due to the capacity contribution from zinc-ion intercalation.

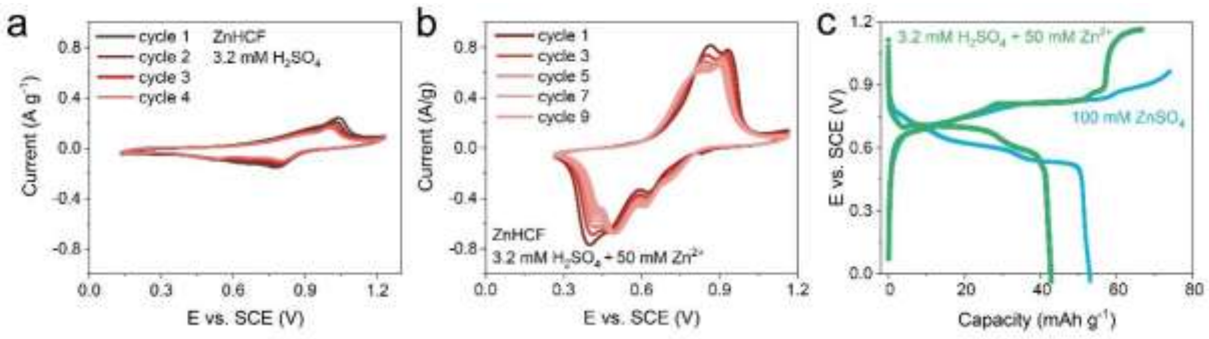

Figure S3. Electrochemical characterization of ZnHCF. (a) Cyclic voltammograms of ZnHCF in 3.2 mM H_2SO_4 solution at 1 mV s^{-1}. (b) Cyclic voltammograms of ZnHCF in 3.2 mM H_2SO_4 solution with 50 mM Zn^{2+} at 1 mV s^{-1}. (c) Galvanostatic charge-discharge profiles of ZnHCF in 3.2 mM H_2SO_4 solution with 50 mM Zn^{2+} (green) and 100 mM $ZnSO_4$ (blue) at 1 C.

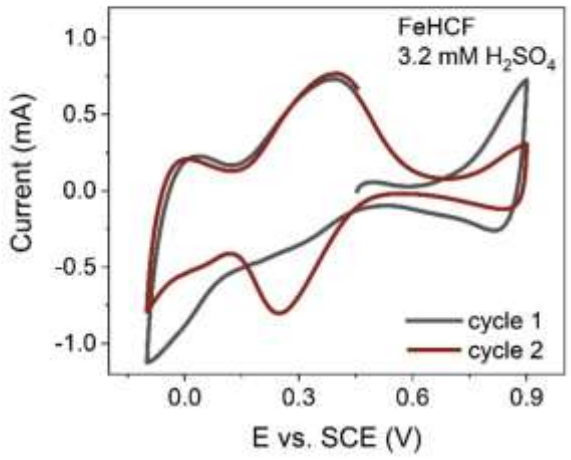

Figure S4. Cyclic voltammograms of FeHCF in 3.2 mM H_2SO_4 solution at 1 mV s^{-1}.

Note S3. M-TBA Stability in acidic solution: theoretical analysis

We calculate the Gibbs free energy change for the M-TBA material dissolution process as follows:

$$HM_{12}[Fe(CN)_6]_8 \cdot 48H_2O + 47\ H^+ \rightarrow 8Fe^{3+} + 48HCN + 12M^{2+} + 48H_2O + e^- \quad (1)$$

To enable the use of the computational hydrogen electrode (CHE) formalism for evaluating the free energy of electron–proton pairs, we add a proton to both sides of Equation (1), resulting in Equation (2):

$$HM_{12}[Fe(CN)_6]_8 \cdot 48H_2O + 48\ H^+ \rightarrow 8Fe^{3+} + 48HCN + 12M^{2+} + 48H_2O + e^- + H^+ \quad (2)$$

The Gibbs free energy change in Equation (2) can be determined using Equation (3):

$$\Delta G_M = 8 * \mu(Fe^{3+}) + 48 * \mu(HCN) + 12 * \mu(M^{2+}) + 48 * \mu(H_2O) + \mu(H^+) + \mu(e^-) - \mu(\text{M-TBA+H}) - 48 * \mu(H^+) \quad (3)$$

We then applied the CHE formalism to calculate $\mu(H^+) + \mu(e^-)$:

$$\mu(H^+) + \mu(e^-) = 0.5 * \mu(H_2, g) - eU_{SHE} - k_BT * \ln 10 * pH \quad (4)$$

Using Equation (4), Equation (3) can be rewritten as shown in Equation (5):

$$\Delta G_M = 8 * \mu(Fe^{3+}) + 48 * \mu(HCN) + 12 * \mu(M^{2+}) + 48 * \mu(H_2O) + 0.5 * \mu(H_2, g) - eU_{SHE} - k_BT * \ln 10 * pH - \mu(\text{M-TBA+H}) - 48 * \mu(H^+) \quad (5)$$

Additionally, we assume that SO_4^{2-} and M^{2+} in solution stay in equilibrium with their salt form in solid, MSO_4 (s). Thus, the chemical potential of M^{2+} was given by $\mu(MSO_4, s) - \mu(SO_4^{2-})$. Therefore, Equation (5) can be rewritten as follows:

$$\Delta G_M = 8 * \mu(Fe^{3+}) + 48 * \mu(HCN) + 12 * \mu(MSO_4, s) - 12 * \mu(SO_4^{2-}) + 48 * \mu(H_2O) + 0.5 * \mu(H_2, g) - eU_{SHE} - k_BT * \ln 10 * pH - \mu(\text{M-TBA+H}) - 48 * \mu(H^+) \quad (6)$$

By rearranging terms in Equation (6) into those related to the metal species M within one bracket and the remaining terms outside the bracket, we arrive at Equation (7):

$$\Delta G_M = [12 * \mu(MSO_{4,}\ s) - \mu(M\text{-}TBA{+}H) - eU_{SHE}] + 8 * \mu(Fe^{3+}) + 48 * \mu(HCN) - 12 * \mu(SO_4^{2-}) + 48 * \mu(H_2O) + 0.5 * \mu(H_2,\ g) - k_BT * \ln 10 * pH - 48 * \mu(H^+) \quad (7)$$

We used ΔG_M for TBA-Cu+H as a reference to cancel out the terms outside the brackets, allowing us to calculate the Gibbs free energy change for M-TBA+H relative to TBA-Cu+H.

Table S1. Values (unit: eV) used in the calculation of the theoretical dissolution rate ratio ($r_{M\text{-}TBA+H}/r_{Cu\text{-}TBA+H}$) (M = Cu, Zn, Mn, Fe, Co, and Ni).

Material	$12*\mu(MSO_{4,}\ s)$ $-\mu(M\text{-}TBA{+}H)$	eU_M	eU_M $-eU_{Cu}$	ΔG_M $-\Delta G_{Cu}$	Theoretical $r_{M\text{-}TBA+H}/r_{Cu\text{-}TBA+H}$
Cu-TBA+H	0.00	1.00	0.00	0.00	1.00
Ni-TBA+H	-0.97	0.72	-0.28	-0.69	4.67E+11
Zn-TBA+H	-1.97	1.52	0.22	-2.19	1.09E+37
Co-TBA+H	-2.60	0.95	-0.15	-2.45	2.72E+41
Fe-TBA+H	-3.32	0.72	-0.28	-3.04	2.59E+51
Mn-TBA+H	-7.86	0.95	-0.05	-7.81	7.74E+131

Note: U_M and U_{Cu} represent the redox potentials for M and Cu-TBA materials, referenced to the standard hydrogen electrode (SHE). ΔG_M and ΔG_{Cu} refer to the Gibbs free energy change of the dissolution of M- and Cu-TBA materials.

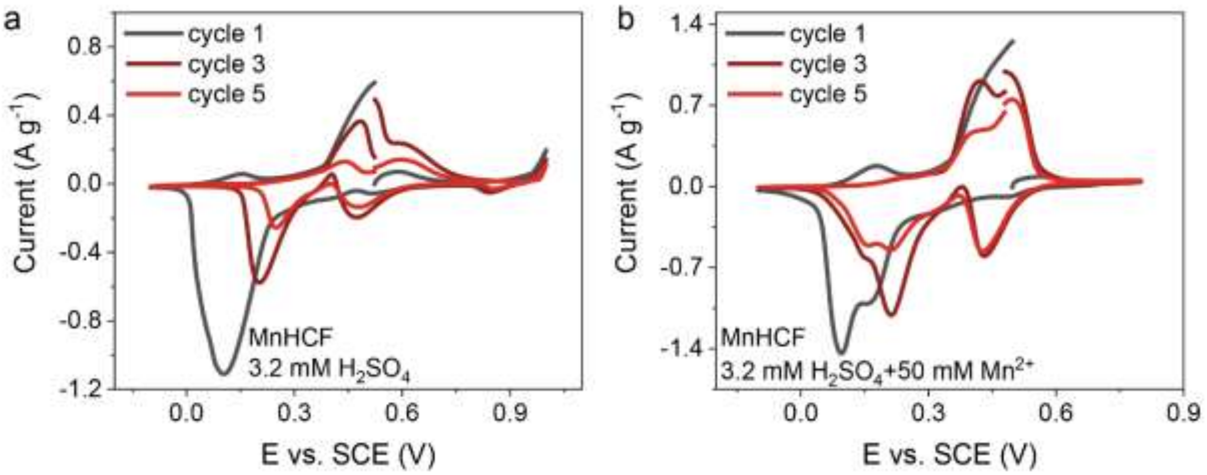

Figure S5. Electrochemical characterization of MnHCF. (a) Cyclic voltammograms of MnHCF in 3.2 mM H_2SO_4 solution at 1 mV s^{-1}. (b) Cyclic voltammograms of MnHCF in 3.2 mM H_2SO_4 solution with 50 mM Mn^{2+} at 1 mV s^{-1}.

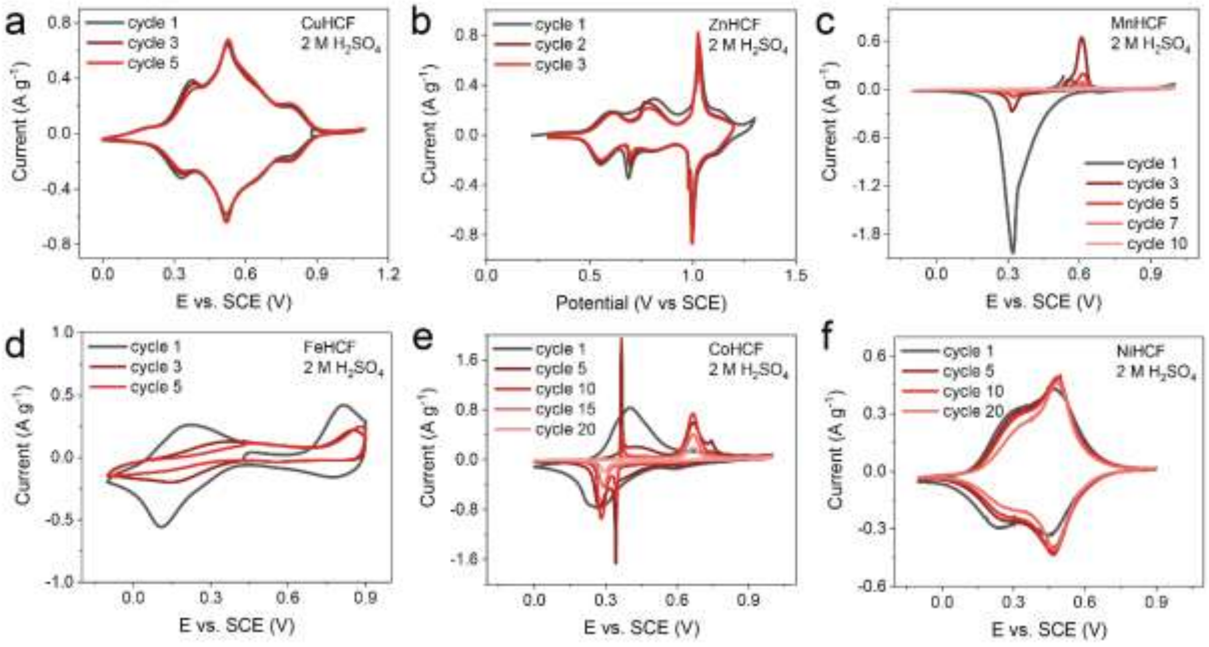

Figure S6. Cyclic voltammograms of various as-synthesized TBAs in 2 M H_2SO_4 at 1 mV s^{-1}. (a) CuHCF. (b) ZnHCF. (c) MnHCF. (d) FeHCF. (e) CoHCF. (f) NiHCF.

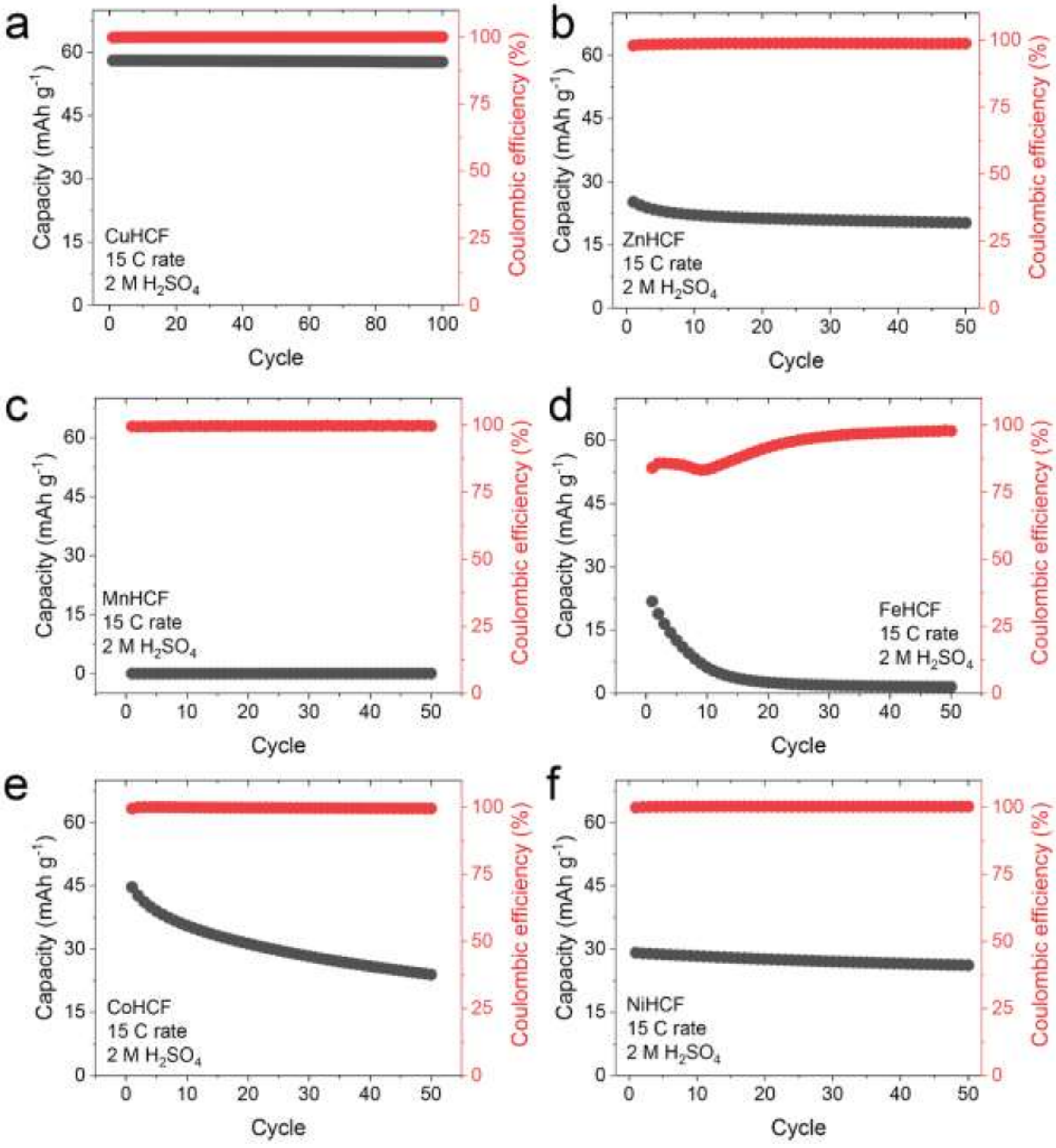

Figure S7. Cyclic stability of various as-synthesized TBAs in 2 M H_2SO_4 at 15 C (1 A g^{-1}). (a) CuHCF. (b) ZnHCF. (c) MnHCF. (d) FeHCF (e) CoHCF. (f) NiHCF. Here, 1 C is 65 mA g^{-1} based on their theoretical capacity.

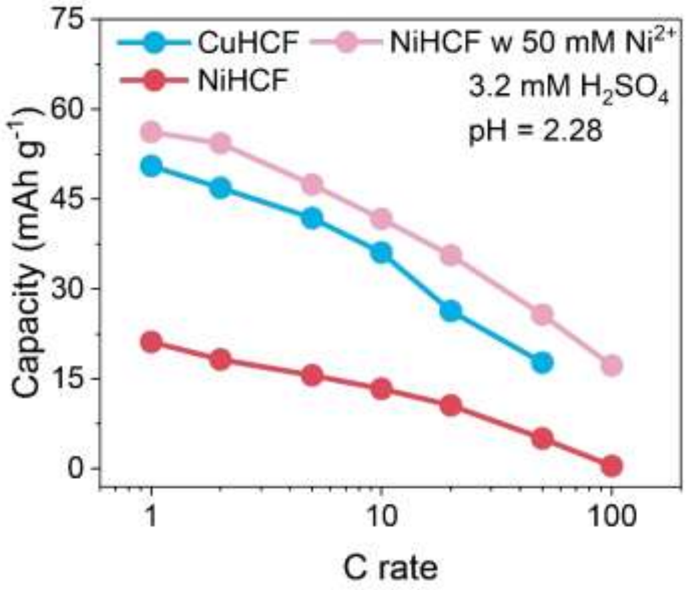

Figure S8. Rate capability of CuHCF and NiHCF in 3.2 mM H_2SO_4. The rate capability of NiHCF was performed in 3.2 mM H_2SO_4 solution (red) and 3.2 mM H_2SO_4 solution with 50 mM Ni^{2+} (pink).

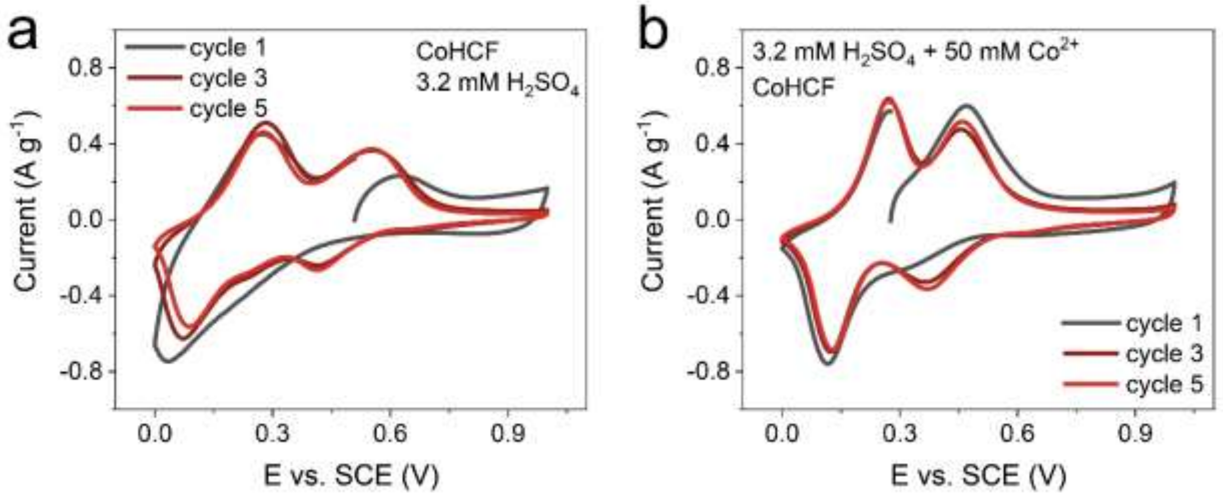

Figure S9. Electrochemical characterization of CoHCF. (a) Cyclic voltammograms of CoHCF in 3.2 mM H_2SO_4 solution at 1 mV s^{-1}. (b) Cyclic voltammograms of CoHCF in 3.2 mM H_2SO_4 solution with 50 mM Co^{2+} at 1 mV s^{-1}.

Note S4. AIMD Evaluation of Cu-TBA Structural Stability at 300 K

Computational Methods

Ab initio molecular dynamics (AIMD)[2–4] simulations were performed to evaluate the finite-temperature stability of the Cu-substituted TBA framework with 48 water molecules. The starting geometry was taken from the fully optimized 0 K DFT structure. The system was first gradually heated from 0 to 300 K over around 3 ps using a Nosé–Hoover thermostat[5,6] with SMASS = 1 and a time step of 0.5 fs, serving only as an equilibration step to bring the system close to the target temperature. Such thermostat parameters and time steps are consistent with those widely employed in previous AIMD studies[7–9]on hydrated and confined-water systems. This rapid-response thermostat enabled efficient ramping of the temperature. During this stage, the instantaneous temperature approached 300 K but had not yet stabilized. A subsequent constant-temperature simulation at 300 K was then carried out using SMASS = 5 and the same 0.5 fs time step. This slower-response thermostat is better suited for production dynamics, avoiding overdamping while preventing spurious resonances with molecular vibrations. Both the heating and production stages were conducted in the NVT ensemble. In Figure S10, t = 0 fs corresponds to the beginning of this constant-temperature stage rather than the heating stage.

The instantaneous temperature and the total energy per atom were monitored along the trajectory. A 50-step moving average (~25 fs) was applied to smooth the raw data. This window length exceeds the fastest vibrational periods of water (OH stretch ~10 fs, HOH bend ~20 fs)[10], thereby filtering out high-frequency oscillations while retaining slower thermodynamic fluctuations. During the steady-state regime, the instantaneous temperature fluctuated around 300 K within the range shown in Figure S10, while the total energy per atom oscillated stably around a constant mean without systematic drift. Such finite-size fluctuations are typical for NVT AIMD simulations

and are comparable to those reported in previous studies of aqueous and confined-water system[8,11]. After equilibration (~2.8 ps into the thermostatting stage), a 5 ps production trajectory was collected, and all frames from this interval were analyzed. The sampling length and equilibration protocol are consistent with previous AIMD studies[7–9], where ~5 ps production windows were adopted once equilibrium was reached. Hydrogen bonds were identified using a conventional geometric criterion: an O·O distance ⩽ 3.5 Å and an O–H·O angle ⩾ 150° [12,13]. For structural deviations of water oxygens, a permutation-invariant root-mean-square deviation (RMSD) was used to account for molecular exchange, as previously applied in molecular dynamics analyses of liquids and cluster ensembles[14,15].

Because AIMD simulations are computationally demanding, certain electronic-structure parameters were slightly relaxed relative to the static DFT settings in order to extend the accessible simulation length. Benchmark tests were carried out on the same Cu-TBA system at the static DFT level to verify that these choices did not affect the accuracy. Reducing the electronic energy convergence threshold from 10^{-6} to 10^{-5} eV changed the total energy by only ~0.02 eV and did not alter the atomic magnetic moments. Similarly, lowering the plane-wave cutoff from 550 to 450 eV resulted in a change of merely ~0.01 eV in the calculated water-framework interaction energy. These benchmarks confirm that the relaxed parameters reproduce the results of the stricter settings, validating their use in AIMD to save computational resources without compromising accuracy. All other parameters, including exchange-correlation functional, PAW potentials, K-point sampling, and initial magnetic moments, were identical to those used in the 0 K DFT calculations.

Results and Discussion

Thermal Stability

During the 300 K stage (sampling window starting at 2805.5 fs, shaded region in Figure S10a), the smoothed temperature fluctuated between approximately 265 and 335 K, which is typical for finite-size NVT simulations at 300 K[8,11] and reflects natural thermal fluctuations rather than instability. The total energy per atom remained nearly constant, varying by only ~0.02 eV (Figure S10b), consistent with fluctuation amplitudes reported for equilibrated AIMD trajectories[16]. The absence of systematic drift in either temperature or energy confirms that the system had reached a thermally stable, steady-state regime. This steady behavior suggests that, once equilibrated near 300 K, the system remains dynamically stable within the accessible AIMD timescale. Subsequent structural analyses therefore compare this equilibrated trajectory against the 0 K DFT reference to evaluate whether the key structural motifs are preserved under ambient conditions.

Radial Distribution Functions

The O-O radial distribution function (RDF) maintains its first-shell peak near ~2.7 Å at both 0 K (red dashed line in Figure S11a) and 300 K (blue solid line in Figure S11a). At 0 K, the peak is sharp and intense, whereas at 300 K it becomes broader and reduced in height. This broadening and amplitude reduction arise from thermal motion of water molecules, which widens the distribution of O-O distances without altering their average geometry. Such temperature-induced broadening has been widely observed in both experimental and simulation studies of liquid water[17]. The O-H (Figure S11b) and H-H (Figure S11c) RDFs show analogous trends: their peak positions remain aligned with the static structure, while amplitudes decrease at finite temperature. These results indicate that the local hydrogen-bond geometry is maintained, with thermal fluctuations merely broadening the distributions rather than causing any structural rearrangement.

Hydrogen Bonding

The static 0 K DFT structure contains 51 hydrogen bonds according to the adopted geometric criterion (red dashed line in Figure S11d). At 300 K, the number fluctuates dynamically between ~40 and 48, with an average of approximately 45. This corresponds to a moderate reduction of ~10-15% compared to the static reference (blue solid line in Figure S11d). With 48 water molecules present, the ratio is close to one hydrogen bond per molecule, which is lower than in bulk liquid water because of the spatial confinement within the framework and the strict angular cutoff used in the definition. Importantly, the hydrogen-bond count remains statistically stable without any monotonic decrease over time, demonstrating that the hydrogen-bond network is preserved and dynamically sustained at room temperature.

Angular Distributions

At 0 K, the O-H···O angle distribution shows multiple sharp peaks clustered within 160-175°, reflecting nearly linear hydrogen bonds in the optimized geometry (red dashed lines in Figure S11e). At 300 K, the distribution broadens into a continuous profile centered around 165-170°, with a tail extending down to ~145° (blue solid lines), indicating that thermal motion induces greater orientational variability. This broadening arises from thermal librations and reorientations of hydrogen bonds, which smear out the discrete low-temperature configurations into a continuous angular spectrum, consistent with previous AIMD analyses of hydrogen-bonded water systems[11]. Despite this broadening, the main angular population remains centered near 165-170°, showing that the preference for near-linear hydrogen bonding is preserved at room temperature.

Structural Deviation Metrics

To further quantify structural fluctuations beyond the RDF and hydrogen-bond analyses, the deviations of water oxygen positions from the 0 K DFT geometry were characterized by a permutation-invariant RMSD (Figure S11f). This definition, introduced earlier, preserves the

indistinguishability of equivalent water molecules by optimally mapping their configurations between frames, thereby eliminating artificial contributions from molecular exchange. The resulting RMSD values remained within ~1.1-1.65 Å throughout the 300 K trajectory, with no systematic drift or monotonic increase. Given that these displacements are substantially smaller than the nearest-neighbor O···O separation (~2.7 Å), they correspond to localized librational and vibrational motions within individual coordination cages rather than molecular diffusion. Therefore, the water molecules exhibit only confined excursions around their equilibrium sites, confirming that the Cu-TBA framework retains its structural integrity under ambient conditions.

Oxygen Occupancy Maps

Two-dimensional occupancy maps projected along the XY (Figure S12a), XZ (Figure S12b), and YZ (Figure S12c) planes provide complementary, real-space evidence of the dynamic oxygen distribution. The occupancy maxima coincide with the DFT oxygen sites, and no additional high-density regions appear, confirming that oxygen atoms remain localized near their crystallographic positions. Although the XZ and YZ projections appear somewhat broader, this effect arises from projection geometry rather than physical diffusion: because the X axis is the longest lattice direction, oxygen sites separated along X are superimposed in these views, leading to apparent elongation in projection. This geometric interpretation is corroborated by the hydrogen-bond and angular analyses (Figs. S11d-e), which show time-stationary statistics and preserved near-linear O–H···O geometries, indicating local librations instead of site exchange. It is further consistent with the permutation-invariant RMSD (Figure S11f), which remains below 2 Å—well within a single coordination cage. Together, these results demonstrate that the broader projected features are artifacts of viewing geometry and thermal librations, not signatures of oxygen migration. The

300 K motion therefore remains confined around equilibrium sites, with no evidence of diffusion or structural rearrangement.

Summary

Across all metrics—temperature and energy stability, RDFs, hydrogen-bond statistics, angle distributions, RMSD, and occupancy maps—the Cu-TBA framework with 48 water molecules retains the essential structural motifs optimized at 0 K when equilibrated at 300 K. Thermal fluctuations broaden the distributions and modestly reduce the hydrogen-bond count by ~10-15%, yet the network topology and site occupancies remain unchanged. No indication of molecular diffusion or framework degradation is observed within the accessible AIMD timescale. These results demonstrate that the Cu-TBA assembly is dynamically robust under ambient conditions, validating the DFT-optimized configuration as a representative equilibrium structure and supporting its use for subsequent mechanistic analyses.

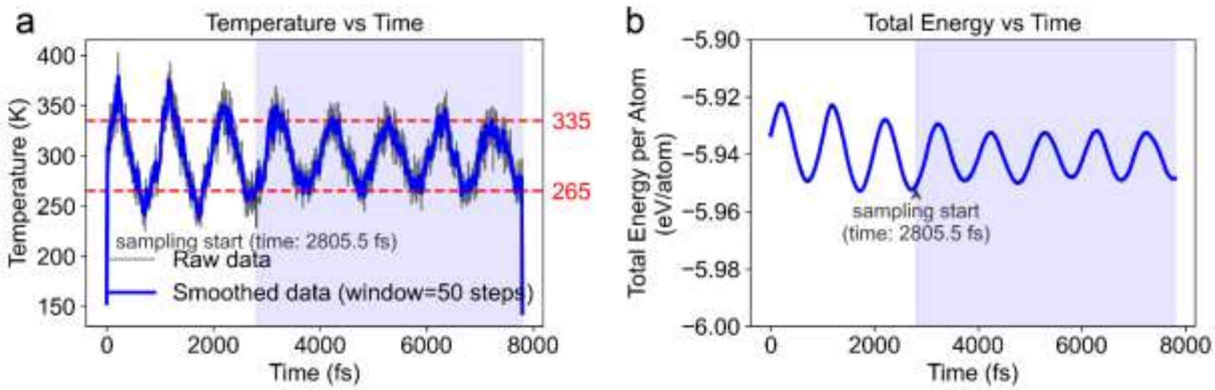

Figure S10. (a) Time evolution of the instantaneous temperature during ab initio molecular dynamics (AIMD) simulations of Cu-TBA with 48 water molecules. The system was first heated from 0 to 300 K over 2 ps using a Nosé-Hoover thermostat (SMASS = 1, time step 0.5 fs), after which a constant-temperature simulation at 300 K was performed using SMASS = 5 and the same time step. The plotted trajectory corresponds to this 300 K stage, and the data were smoothed using a 50-step moving average (~25 fs) to remove high-frequency vibrational noise. The shaded region marks the ~5 ps steady-state window used for structural analysis, beginning once the temperature and total energy had stabilized. During this stage, the smoothed temperature fluctuated within 265-335 K (±35 K around the target). (b) Time evolution of the total energy per atom during the same simulation. The shaded region again corresponds to the ~5 ps production window, where the system is in steady-state regime. Within this interval, the energy fluctuates within ~0.02 eV per atom around a constant mean, consistent with the thermal stability shown in panel (a).

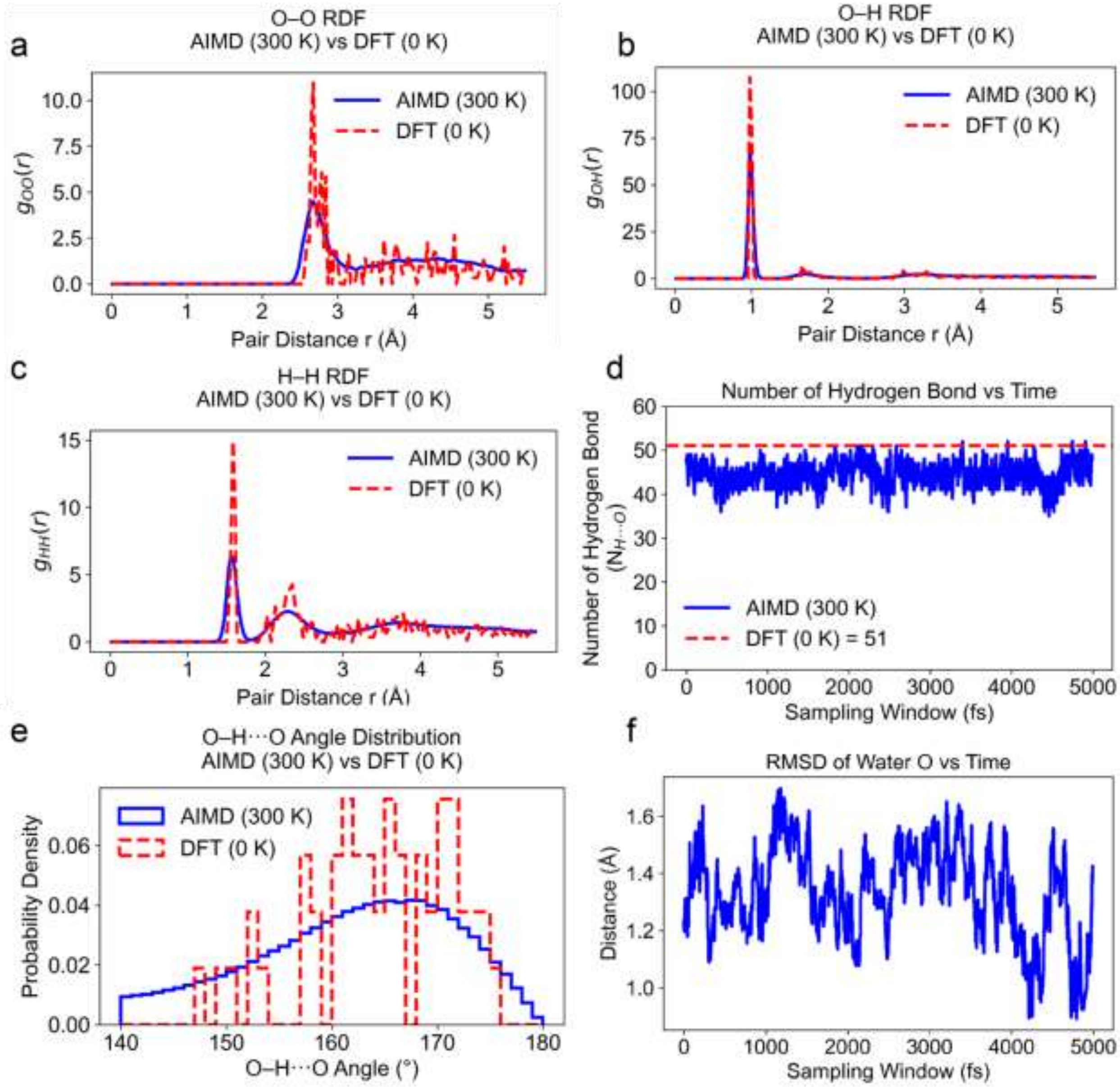

Figure S11. (a-c) Radial distribution functions (RDFs) for O-O, O-H, and H-H pairs in Cu-TBA, comparing the optimized 0 K DFT structure with the ab initio molecular dynamics (AIMD) trajectory at 300 K. (d) Time evolution of the number of hydrogen bonds during the 300 K AIMD trajectory, identified using the geometric criterion O·O $\leqslant$ 3.5 Å and O–H·O $\geqslant$ 150°. The shaded region marks the ~5 ps production window used for statistical analysis. (e) O–H···O angle distribution, comparing the optimized 0 K DFT structure with the AIMD trajectory at 300 K. Thermal librations broaden the angular distribution but the preference for near-linear geometries is maintained. (f) Permutation-invariant root-mean-square deviation (RMSD) of water-oxygen positions relative to the 0 K DFT reference, computed over the same 300 K production window. This definition accounts for molecular indistinguishability and exchange, ensuring that the RMSD reflects genuine structural fluctuations rather than artificial reindexing effects.

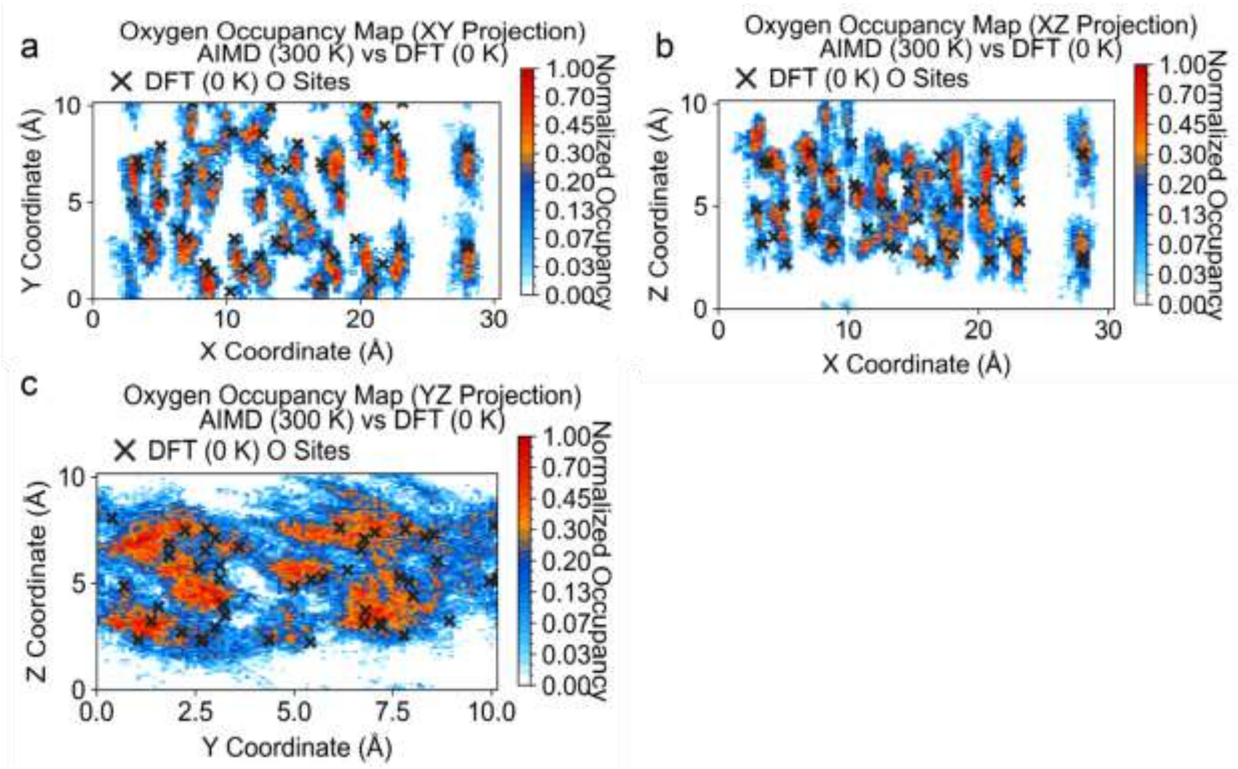

Figure S12. Two-dimensional occupancy maps of water-oxygen positions projected along the (a) XY, (b) XZ, and (c) YZ planes. Black crosses mark the static oxygen sites from the 0 K DFT structure, while the blue density corresponds to the ab initio molecular dynamics (AIMD) trajectory at 300 K. The maxima of the occupancy distributions coincide with the DFT oxygen sites, indicating that the water molecules remain localized near their equilibrium positions. The apparently broader features in the XZ and YZ projections arise from the superposition of distinct oxygen sites along the elongated X direction, rather than from physical diffusion.

Note S5. Bond-Length Analysis and Local Coordination Distortions in IS and FS Geometries

Table S2. Average bond lengths (M-N, Fe-C) in the optimized IS and FS geometries and their differences (unit: Å).

Material	d_{C-Fe}^{IS}	d_{C-Fe}^{FS}	Δd_{C-Fe}^{FS-IS}	d_{N-M}^{IS}	d_{N-M}^{FS}	Δd_{N-M}^{FS-IS}
Cu-TBA+H	1.92	1.92	0.00	3.22	3.22	0.00
Ni-TBA+H	1.92	1.92	0.00	3.28	3.28	0.00
Zn-TBA+H	1.91	1.91	0.00	3.24	3.25	0.01
Co-TBA+H	1.91	1.91	0.00	3.28	3.28	0.00
Fe-TBA+H	1.90	1.90	0.00	3.29	3.28	-0.01
Mn-TBA+H	1.90	1.90	0.00	3.32	3.33	0.01

Note: $d_{C-Fe}^{IS(FS)}$ and $d_{N-M}^{IS(FS)}$ correspond to the average Fe-C and M-N distances in the optimized IS (FS) geometries. M denotes the metal coordinated to the cyanide N atoms.

Table S3. Bond-length spreads ($\delta d = d_{\max} - d_{\min}$) for the optimized IS and FS geometries (unit: Å).

Material	δd_{C-Fe}^{IS}	δd_{C-Fe}^{FS}	$\Delta\delta d_{C-Fe}^{FS-IS}$	δd_{N-M}^{IS}	δd_{N-M}^{FS}	$\Delta\delta d_{N-M}^{FS-IS}$
Cu-TBA+H	0.09	0.09	0.00	3.95	4.05	0.10
Ni-TBA+H	0.07	0.07	0.00	3.98	3.95	-0.03
Zn-TBA+H	0.09	0.09	0.00	4.09	4.05	-0.04
Co-TBA+H	0.08	0.08	0.00	3.84	4.10	0.26
Fe-TBA+H	0.07	0.07	0.00	3.92	3.93	0.01
Mn-TBA+H	0.09	0.08	-0.01	3.75	3.84	-0.09

Note: $\delta d_{C-Fe}^{IS(FS)}$ and $\delta d_{N-M}^{IS(FS)}$ denote the bond-length spreads for Fe-C and M-N coordination environments in the optimized IS (FS) geometries. M denotes the metal coordinated to the cyanide N atoms.

Reference cited in the Supporting Information

(1) Zhang, L.; Chen, L.; Zhou, X.; Liu, Z. Morphology-Dependent Electrochemical Performance of Zinc Hexacyanoferrate Cathode for Zinc-Ion Battery. *Sci. Rep.* **2015**, *5* (1), 18263.

(2) Payne, M. C.; Teter, M. P.; Allan, D. C.; Arias, T. A.; Joannopoulos, J. D. Iterative Minimization Techniques for Ab Initio Total-Energy Calculations: Molecular Dynamics and Conjugate Gradients. *Rev. Mod. Phys.* **1992**, *64* (4), 1045–1097.

(3) Car, R.; Parrinello, M. Unified Approach for Molecular Dynamics and Density-Functional Theory. *Phys. Rev. Lett.* **1985**, *55* (22), 2471–2474.

(4) Tuckerman, M. E. Ab Initio Molecular Dynamics: Basic Concepts, Current Trends and Novel. ***J.** Phys. Condens. Matter* **2002**, *14* (50), R1297.

(5) Nosé, S. A Unified Formulation of the Constant Temperature Molecular Dynamics Methods. *J. Chem. Phys.* **1984**, *81* (1), 511–519.

(6) Nosé, S. A Molecular Dynamics Method for Simulations in the Canonical Ensemble. *Mol. Phys.* **1984**, *52* (2), 255–268.

(7) Wang, Y.; Wang, S.; Fu, Y.; Sang, J.; Wei, P.; Li, R.; Gao, D.; Wang, G.; Bao, X. Ammonia Electrosynthesis from Nitrate Using a Stable Amorphous/Crystalline Dual-Phase Cu Catalyst. *Nat. Commun.* **2025**, *16* (1), 897.

(8) Sun, K.; Wu, X.; Zhuang, Z.; Liu, L.; Fang, J.; Zeng, L.; Ma, J.; Liu, S.; Li, J.; Dai, R.; Tan, X.; Yu, K.; Liu, D.; Cheong, W. C.; Huang, A.; Liu, Y.; Pan, Y.; Xiao, H.; Chen, C. Interfacial Water Engineering Boosts Neutral Water Reduction. *Nat. Commun.* **2022**, *13* (1), 6260.

(9) Ding, Z.; Selloni, A. Modeling the Aqueous Interface of Amorphous TiO_2 Using Deep Potential Molecular Dynamics. *J. Chem. Phys.* **2023**, *159* (2), 024706.

(10) Herzberg, G.; Crawford, B. L. Jr. Infrared and Raman Spectra of Polyatomic Molecules. *J. Phys. Chem.* **1946**, *50* (3), 288.

(11) Moustafa, S. G.; Schultz, A. J.; Kofke, D. A. Effects of Thermostatting in Molecular Dynamics on Anharmonic Properties of Crystals: Application to Fcc Al at High Pressure and Temperature. *J. Chem. Phys.* **2018**, *149* (12), 124109.

(12) Chandra, A.; Chowdhuri, S. Effects of Hydrogen-Bond Environment on Single Particle and Pair Dynamics in Liquid Water. *Proc. Indian Acad. Sci. (Chem. Sci.)* **2001**, *113* (5-6), 591-601.

(13) Kumar, R.; Schmidt, J. R.; Skinner, J. L. Hydrogen Bonding Definitions and Dynamics in Liquid Water. *J. Chem. Phys.* **2007**, *126* (20), 204107.

(14) Pracht, P. Conformational Pruning via the Permutation Invariant Root Mean Square Deviation of Atomic Positions. *J. Chem. Inf. Model.* **2024**, *65* (9), 4501-4511.

(15) Ferré, G.; Maillet, J. B.; Stoltz, G. Permutation-Invariant Distance between Atomic Configurations. *J. Chem. Phys.* **2015**, *143* (10), 104114.

(16) Soper, A. K. The Radial Distribution Functions of Water and Ice from 220 to 673 K and at Pressures up to 400 MPa. *Chem. Phys.* **2000**, *258* (2-3), 121-137.
